# Bringing metabolic networks to life: convenience rate law and thermodynamic constraints

**DOI:** 10.1186/1742-4682-3-41

**Published:** 2006-12-15

**Authors:** Wolfram Liebermeister, Edda Klipp

**Affiliations:** 1 Computational Systems Biology, Max Planck Institute for Molecular Genetics, Ihnestraße 63-73, 14195 Berlin, Germany

## Abstract

**Background:**

Translating a known metabolic network into a dynamic model requires rate laws for all chemical reactions. The mathematical expressions depend on the underlying enzymatic mechanism; they can become quite involved and may contain a large number of parameters. Rate laws and enzyme parameters are still unknown for most enzymes.

**Results:**

We introduce a simple and general rate law called "convenience kinetics". It can be derived from a simple random-order enzyme mechanism. Thermodynamic laws can impose dependencies on the kinetic parameters. Hence, to facilitate model fitting and parameter optimisation for large networks, we introduce thermodynamically independent system parameters: their values can be varied independently, without violating thermodynamical constraints. We achieve this by expressing the equilibrium constants either by Gibbs free energies of formation or by a set of independent equilibrium constants. The remaining system parameters are mean turnover rates, generalised Michaelis-Menten constants, and constants for inhibition and activation. All parameters correspond to molecular energies, for instance, binding energies between reactants and enzyme.

**Conclusion:**

Convenience kinetics can be used to translate a biochemical network – manually or automatically - into a dynamical model with plausible biological properties. It implements enzyme saturation and regulation by activators and inhibitors, covers all possible reaction stoichiometries, and can be specified by a small number of parameters. Its mathematical form makes it especially suitable for parameter estimation and optimisation. Parameter estimates can be easily computed from a least-squares fit to Michaelis-Menten values, turnover rates, equilibrium constants, and other quantities that are routinely measured in enzyme assays and stored in kinetic databases.

## Background

Dynamic modelling of biochemical networks requires quantitative information about enzymatic reactions. Because many metabolic networks are known and stored in databases [1,2], it would be desirable to translate networks automatically into kinetic models that are in agreement with the available data. As a first attempt, all reactions could be described by versatile laws such as mass-action kinetics, generalised mass-action kinetics [3,4] or linlog kinetics [5,6]. However, these kinetic laws fail to describe enzyme saturation at high substrate concentrations, which is a common and relevant phenomenon.

A prominent example of a saturable kinetics is the reversible form of the traditional Michaelis-Menten kinetics [7] for a reaction *A *↔ *B*. At substrate concentration *a *and product concentration *b *(measured in mM), the reaction rate reads

v(a,b)=Ek+cata˜−k−catb˜1+a˜+b˜     (1)
 MathType@MTEF@5@5@+=feaafiart1ev1aaatCvAUfKttLearuWrP9MDH5MBPbIqV92AaeXatLxBI9gBaebbnrfifHhDYfgasaacH8akY=wiFfYdH8Gipec8Eeeu0xXdbba9frFj0=OqFfea0dXdd9vqai=hGuQ8kuc9pgc9s8qqaq=dirpe0xb9q8qiLsFr0=vr0=vr0dc8meaabaqaciaacaGaaeqabaqabeGadaaakeaacqWG2bGDcqGGOaakcqWGHbqycqGGSaalcqWGIbGycqGGPaqkcqGH9aqpcqWGfbqrdaWcaaqaaiabdUgaRnaaDaaaleaacqGHRaWkaeaaieaacqWFJbWycqWFHbqycqWF0baDaaGccuWGHbqygaacaiabgkHiTiabdUgaRnaaDaaaleaacqGHsislaeaacqWFJbWycqWFHbqycqWF0baDaaGccuWGIbGygaacaaqaaiabigdaXiabgUcaRiqbdggaHzaaiaGaey4kaSIafmOyaiMbaGaaaaGaaCzcaiaaxMaadaqadaqaaiabigdaXaGaayjkaiaawMcaaaaa@4F40@

with enzyme concentration *E*, turnover rates k+cat
 MathType@MTEF@5@5@+=feaafiart1ev1aaatCvAUfKttLearuWrP9MDH5MBPbIqV92AaeXatLxBI9gBaebbnrfifHhDYfgasaacH8akY=wiFfYdH8Gipec8Eeeu0xXdbba9frFj0=OqFfea0dXdd9vqai=hGuQ8kuc9pgc9s8qqaq=dirpe0xb9q8qiLsFr0=vr0=vr0dc8meaabaqaciaacaGaaeqabaqabeGadaaakeaacqWGRbWAdaqhaaWcbaGaey4kaScabaacbaGae83yamMae8xyaeMae8hDaqhaaaaa@3322@ and k−cat
 MathType@MTEF@5@5@+=feaafiart1ev1aaatCvAUfKttLearuWrP9MDH5MBPbIqV92AaeXatLxBI9gBaebbnrfifHhDYfgasaacH8akY=wiFfYdH8Gipec8Eeeu0xXdbba9frFj0=OqFfea0dXdd9vqai=hGuQ8kuc9pgc9s8qqaq=dirpe0xb9q8qiLsFr0=vr0=vr0dc8meaabaqaciaacaGaaeqabaqabeGadaaakeaacqWGRbWAdaqhaaWcbaGaeyOeI0cabaacbaGae83yamMae8xyaeMae8hDaqhaaaaa@332D@ (measured in s^-1^), the shortcuts *ã *= *a*/kaM
 MathType@MTEF@5@5@+=feaafiart1ev1aaatCvAUfKttLearuWrP9MDH5MBPbIqV92AaeXatLxBI9gBaebbnrfifHhDYfgasaacH8akY=wiFfYdH8Gipec8Eeeu0xXdbba9frFj0=OqFfea0dXdd9vqai=hGuQ8kuc9pgc9s8qqaq=dirpe0xb9q8qiLsFr0=vr0=vr0dc8meaabaqaciaacaGaaeqabaqabeGadaaakeaacqWGRbWAdaqhaaWcbaacbaGae8xyaegabaGae8xta0eaaaaa@30A7@ and b˜
 MathType@MTEF@5@5@+=feaafiart1ev1aaatCvAUfKttLearuWrP9MDH5MBPbIqV92AaeXatLxBI9gBaebbnrfifHhDYfgasaacH8akY=wiFfYdH8Gipec8Eeeu0xXdbba9frFj0=OqFfea0dXdd9vqai=hGuQ8kuc9pgc9s8qqaq=dirpe0xb9q8qiLsFr0=vr0=vr0dc8meaabaqaciaacaGaaeqabaqabeGadaaakeaacuWGIbGygaacaaaa@2E08@ = *b*/kbM
 MathType@MTEF@5@5@+=feaafiart1ev1aaatCvAUfKttLearuWrP9MDH5MBPbIqV92AaeXatLxBI9gBaebbnrfifHhDYfgasaacH8akY=wiFfYdH8Gipec8Eeeu0xXdbba9frFj0=OqFfea0dXdd9vqai=hGuQ8kuc9pgc9s8qqaq=dirpe0xb9q8qiLsFr0=vr0=vr0dc8meaabaqaciaacaGaaeqabaqabeGadaaakeaacqWGRbWAdaqhaaWcbaacbaGae8NyaigabaGae8xta0eaaaaa@30A9@, and Michaelis-Menten constants kaM
 MathType@MTEF@5@5@+=feaafiart1ev1aaatCvAUfKttLearuWrP9MDH5MBPbIqV92AaeXatLxBI9gBaebbnrfifHhDYfgasaacH8akY=wiFfYdH8Gipec8Eeeu0xXdbba9frFj0=OqFfea0dXdd9vqai=hGuQ8kuc9pgc9s8qqaq=dirpe0xb9q8qiLsFr0=vr0=vr0dc8meaabaqaciaacaGaaeqabaqabeGadaaakeaacqWGRbWAdaqhaaWcbaacbaGae8xyaegabaGae8xta0eaaaaa@30A7@ and kbM
 MathType@MTEF@5@5@+=feaafiart1ev1aaatCvAUfKttLearuWrP9MDH5MBPbIqV92AaeXatLxBI9gBaebbnrfifHhDYfgasaacH8akY=wiFfYdH8Gipec8Eeeu0xXdbba9frFj0=OqFfea0dXdd9vqai=hGuQ8kuc9pgc9s8qqaq=dirpe0xb9q8qiLsFr0=vr0=vr0dc8meaabaqaciaacaGaaeqabaqabeGadaaakeaacqWGRbWAdaqhaaWcbaacbaGae8NyaigabaGae8xta0eaaaaa@30A9@ (in mM). The rate law (1) can be derived from an enzyme mechanism: kaM
 MathType@MTEF@5@5@+=feaafiart1ev1aaatCvAUfKttLearuWrP9MDH5MBPbIqV92AaeXatLxBI9gBaebbnrfifHhDYfgasaacH8akY=wiFfYdH8Gipec8Eeeu0xXdbba9frFj0=OqFfea0dXdd9vqai=hGuQ8kuc9pgc9s8qqaq=dirpe0xb9q8qiLsFr0=vr0=vr0dc8meaabaqaciaacaGaaeqabaqabeGadaaakeaacqWGRbWAdaqhaaWcbaacbaGae8xyaegabaGae8xta0eaaaaa@30A7@ and kbM
 MathType@MTEF@5@5@+=feaafiart1ev1aaatCvAUfKttLearuWrP9MDH5MBPbIqV92AaeXatLxBI9gBaebbnrfifHhDYfgasaacH8akY=wiFfYdH8Gipec8Eeeu0xXdbba9frFj0=OqFfea0dXdd9vqai=hGuQ8kuc9pgc9s8qqaq=dirpe0xb9q8qiLsFr0=vr0=vr0dc8meaabaqaciaacaGaaeqabaqabeGadaaakeaacqWGRbWAdaqhaaWcbaacbaGae8NyaigabaGae8xta0eaaaaa@30A9@ are the dissociation constants for reactants bound to the enzyme. In the original work by Michaelis and Menten for irreversible kinetics, *k*^M ^was a dissociation constant. Later, Briggs and Haldane presented a different derivation that assumes a quasi-steady state for the enzyme-substrate complex and defines *k*^M ^as the sum of rate constants for complex degradation, divided by the rate constant for complex production, *k*^M ^= (*k*_-1 _+ *k*_2_)/*k*_1_. Other kinetic laws have been derived from specific molecular reaction mechanisms [8,9]; they can have complicated mathematical forms and have to be established separately for each reaction stoichiometry.

Large numbers of enzyme kinetic parameters, such as equilibrium constants, Michaelis-Menten values, turnover rates, or inhibition constants have been collected in databases [10-12], but using them for modelling is not at all straightforward: the values have usually been measured under different, often in-vitro conditions, so they may be incompatible with each other or inappropriate for a certain model [13,14]. In addition, the second law of thermodynamics implies constraints between the kinetic parameters: in a metabolic system, the Gibbs free energies of formation of the metabolites determine the equilibrium constants of the reactions [15]. This leads to constraints between kinetic parameters within reactions [16] and across the entire network [17,18] – a big disadvantage for all methods that scan the parameter space, such as parameter fitting, sampling, and optimisation. Also, if parameter values are guessed from experiments and then directly inserted into a model, this model is likely to be thermodynamically wrong.

We describe here a saturable rate law which we call "convenience kinetics" owing to its favourable properties: it is a generalised form of Michaelis-Menten kinetics, covers all possible stoichiometries, describes enzyme regulation by activators and inhibitors, and can be derived from a rapid-equilibrium random-order enzyme mechanism. To ensure thermodynamic correctness, we write the convenience kinetics in terms of thermodynamically independent parameters [18]. A short introduction to kinetic modelling is given in the methods section; a list of mathematical symbols and an illustrative example is also provided [See Additional file 1]. The companion article [19] explains how the parameters can be estimated from an integration of thermodynamic, kinetic, metabolic, and proteomic data.

## Results and discussion

### The convenience kinetics

The simple form of equation (1) encourages us to use a similar formula for other stoichiometries. For a reaction

*A*_1 _+ *A*_2 _+ ... ↔ *B*_1 _+ *B*_2 _+ ...

with concentration vectors **a **= (*a*_1_, *a*_2_, ...)^T ^and **b **= (*b*_1_, *b*_2_, ...)^T^, we define the convenience kinetics

v(a,b)=Ek+cat∏ia˜i−k−cat∏jb˜j∏i(1+a˜i)+∏j(1+b˜j)−1.     (2)
 MathType@MTEF@5@5@+=feaafiart1ev1aaatCvAUfKttLearuWrP9MDH5MBPbIqV92AaeXatLxBI9gBaebbnrfifHhDYfgasaacH8akY=wiFfYdH8Gipec8Eeeu0xXdbba9frFj0=OqFfea0dXdd9vqai=hGuQ8kuc9pgc9s8qqaq=dirpe0xb9q8qiLsFr0=vr0=vr0dc8meaabaqaciaacaGaaeqabaqabeGadaaakeaacqWG2bGDcqGGOaakieqacqWFHbqycqGGSaalcqWFIbGycqGGPaqkcqGH9aqpcqWGfbqrdaWcaaqaaiabdUgaRnaaDaaaleaacqGHRaWkaeaaieaacqGFJbWycqGFHbqycqGF0baDaaGcdaqeqbqaaiqbdggaHzaaiaWaaSbaaSqaaiabdMgaPbqabaaabaGaemyAaKgabeqdcqGHpis1aOGaeyOeI0Iaem4AaS2aa0baaSqaaiabgkHiTaqaaiab+ngaJjab+fgaHjab+rha0baakmaarafabaGafmOyaiMbaGaadaWgaaWcbaGaemOAaOgabeaaaeaacqWGQbGAaeqaniabg+GivdaakeaadaqeqbqaaiabcIcaOiabigdaXiabgUcaRiqbdggaHzaaiaWaaSbaaSqaaiabdMgaPbqabaGccqGGPaqkaSqaaiabdMgaPbqab0Gaey4dIunakiabgUcaRmaarafabaGaeiikaGIaeGymaeJaey4kaSIafmOyaiMbaGaadaWgaaWcbaGaemOAaOgabeaakiabcMcaPaWcbaGaemOAaOgabeqdcqGHpis1aOGaeyOeI0IaeGymaedaaiabc6caUiaaxMaacaWLjaWaaeWaaeaacqaIYaGmaiaawIcacaGLPaaaaaa@6B33@

By analogy to the *k*^M ^values in Michaelis-Menten kinetics, we have defined substrate constants kaiM
 MathType@MTEF@5@5@+=feaafiart1ev1aaatCvAUfKttLearuWrP9MDH5MBPbIqV92AaeXatLxBI9gBaebbnrfifHhDYfgasaacH8akY=wiFfYdH8Gipec8Eeeu0xXdbba9frFj0=OqFfea0dXdd9vqai=hGuQ8kuc9pgc9s8qqaq=dirpe0xb9q8qiLsFr0=vr0=vr0dc8meaabaqaciaacaGaaeqabaqabeGadaaakeaacqWGRbWAdaqhaaWcbaacbaGae8xyae2aaSbaaWqaaiab=LgaPbqabaaaleaacqWFnbqtaaaaaa@3236@ and product constants kbjM
 MathType@MTEF@5@5@+=feaafiart1ev1aaatCvAUfKttLearuWrP9MDH5MBPbIqV92AaeXatLxBI9gBaebbnrfifHhDYfgasaacH8akY=wiFfYdH8Gipec8Eeeu0xXdbba9frFj0=OqFfea0dXdd9vqai=hGuQ8kuc9pgc9s8qqaq=dirpe0xb9q8qiLsFr0=vr0=vr0dc8meaabaqaciaacaGaaeqabaqabeGadaaakeaacqWGRbWAdaqhaaWcbaacbaGae8Nyai2aaSbaaWqaaiab=PgaQbqabaaaleaacqWFnbqtaaaaaa@323A@ (in mM); just as above, variables with a tilde denote the normalised reactant concentrations *ã*_*i *_= *a*_*i*_/kaiM
 MathType@MTEF@5@5@+=feaafiart1ev1aaatCvAUfKttLearuWrP9MDH5MBPbIqV92AaeXatLxBI9gBaebbnrfifHhDYfgasaacH8akY=wiFfYdH8Gipec8Eeeu0xXdbba9frFj0=OqFfea0dXdd9vqai=hGuQ8kuc9pgc9s8qqaq=dirpe0xb9q8qiLsFr0=vr0=vr0dc8meaabaqaciaacaGaaeqabaqabeGadaaakeaacqWGRbWAdaqhaaWcbaacbaGae8xyae2aaSbaaWqaaiab=LgaPbqabaaaleaacqWFnbqtaaaaaa@3236@ and b˜
 MathType@MTEF@5@5@+=feaafiart1ev1aaatCvAUfKttLearuWrP9MDH5MBPbIqV92AaeXatLxBI9gBaebbnrfifHhDYfgasaacH8akY=wiFfYdH8Gipec8Eeeu0xXdbba9frFj0=OqFfea0dXdd9vqai=hGuQ8kuc9pgc9s8qqaq=dirpe0xb9q8qiLsFr0=vr0=vr0dc8meaabaqaciaacaGaaeqabaqabeGadaaakeaacuWGIbGygaacaaaa@2E08@_*j *_= *b*_*j*_/kbjM
 MathType@MTEF@5@5@+=feaafiart1ev1aaatCvAUfKttLearuWrP9MDH5MBPbIqV92AaeXatLxBI9gBaebbnrfifHhDYfgasaacH8akY=wiFfYdH8Gipec8Eeeu0xXdbba9frFj0=OqFfea0dXdd9vqai=hGuQ8kuc9pgc9s8qqaq=dirpe0xb9q8qiLsFr0=vr0=vr0dc8meaabaqaciaacaGaaeqabaqabeGadaaakeaacqWGRbWAdaqhaaWcbaacbaGae8Nyai2aaSbaaWqaaiab=PgaQbqabaaaleaacqWFnbqtaaaaaa@323A@. If the denominator is multiplied out, it contains all mathematical products of normalised substrate concentrations and product concentrations, but no mixed terms containing substrates and products together; the term +1 in the denominator is supposed to appear only once, so it is subtracted in the end. If several molecules of the same substance participate in a reaction, that is, for general stoichiometries

*α*_1 _*A*_1 _+ *α*_2 _*A*_2 _+ ... ↔ *β*_1 _*B*_1 _+ *β*_2 _*B*_2 _+ ...,

the formula looks slightly different:

v(a,b)=Ek+cat∏ia˜iαi−k−cat∏jb˜jβj∏i(1+a˜i+...+a˜iαi)+∏j(1+b˜j+...+b˜jβj)−1=Ek+cat∏ia˜iαi−k−cat∏jb˜jβj∏i(∑m=0αi(a˜i)m)+∏j(∑m=0βj(b˜j)m)−1.     (3)
 MathType@MTEF@5@5@+=feaafiart1ev1aaatCvAUfKttLearuWrP9MDH5MBPbIqV92AaeXatLxBI9gBaebbnrfifHhDYfgasaacH8akY=wiFfYdH8Gipec8Eeeu0xXdbba9frFj0=OqFfea0dXdd9vqai=hGuQ8kuc9pgc9s8qqaq=dirpe0xb9q8qiLsFr0=vr0=vr0dc8meaabaqaciaacaGaaeqabaqabeGadaaakeaafaqadeGadaaabaGaemODayNaeiikaGccbeGae8xyaeMaeiilaWIae8NyaiMaeiykaKcabaGaeyypa0dabaGaemyrau0aaSaaaeaacqWGRbWAdaqhaaWcbaGaey4kaScabaacbaGae43yamMae4xyaeMae4hDaqhaaOWaaebuaeaacuWGHbqygaacamaaDaaaleaacqWGPbqAaeaaiiGacqqFXoqydaWgaaadbaGaemyAaKgabeaaaaaaleaacqWGPbqAaeqaniabg+GivdGccqGHsislcqWGRbWAdaqhaaWcbaGaeyOeI0cabaGae43yamMae4xyaeMae4hDaqhaaOWaaebuaeaacuWGIbGygaacamaaDaaaleaacqWGQbGAaeaacqqFYoGydaWgaaadbaGaemOAaOgabeaaaaaaleaacqWGQbGAaeqaniabg+GivdaakeaadaqeqbqaaiabcIcaOiabigdaXiabgUcaRiqbdggaHzaaiaWaaSbaaSqaaiabdMgaPbqabaGccqGHRaWkcqGGUaGlcqGGUaGlcqGGUaGlcqGHRaWkcuWGHbqygaacamaaDaaaleaacqWGPbqAaeaacqqFXoqydaWgaaadbaGaemyAaKgabeaaaaGccqGGPaqkaSqaaiabdMgaPbqab0Gaey4dIunakiabgUcaRmaarafabaGaeiikaGIaeGymaeJaey4kaSIafmOyaiMbaGaadaWgaaWcbaGaemOAaOgabeaakiabgUcaRiabc6caUiabc6caUiabc6caUiabgUcaRiqbdkgaIzaaiaWaa0baaSqaaiabdQgaQbqaaiab9j7aInaaBaaameaacqWGQbGAaeqaaaaakiabcMcaPaWcbaGaemOAaOgabeqdcqGHpis1aOGaeyOeI0IaeGymaedaaaqaaaqaaiabg2da9aqaaiabdweafnaalaaabaGaem4AaS2aa0baaSqaaiabgUcaRaqaaiab+ngaJjab+fgaHjab+rha0baakmaarafabaGafmyyaeMbaGaadaqhaaWcbaGaemyAaKgabaGae0xSde2aaSbaaWqaaiabdMgaPbqabaaaaaWcbaGaemyAaKgabeqdcqGHpis1aOGaeyOeI0Iaem4AaS2aa0baaSqaaiabgkHiTaqaaiab+ngaJjab+fgaHjab+rha0baakmaarafabaGafmOyaiMbaGaadaqhaaWcbaGaemOAaOgabaGae0NSdi2aaSbaaWqaaiabdQgaQbqabaaaaaWcbaGaemOAaOgabeqdcqGHpis1aaGcbaWaaebuaeaacqGGOaakdaaeWbqaaiabcIcaOiqbdggaHzaaiaWaaSbaaSqaaiabdMgaPbqabaGccqGGPaqkdaahaaWcbeqaaiabd2gaTbaakiabcMcaPaWcbaGaemyBa0Maeyypa0JaeGimaadabaGae0xSde2aaSbaaWqaaiabdMgaPbqabaaaniabggHiLdaaleaacqWGPbqAaeqaniabg+GivdGccqGHRaWkdaqeqbqaaiabcIcaOmaaqahabaGaeiikaGIafmOyaiMbaGaadaWgaaWcbaGaemOAaOgabeaakiabcMcaPmaaCaaaleqabaGaemyBa0gaaOGaeiykaKcaleaacqWGTbqBcqGH9aqpcqaIWaamaeaacqqFYoGydaWgaaadbaGaemOAaOgabeaaa0GaeyyeIuoaaSqaaiabdQgaQbqab0Gaey4dIunakiabgkHiTiabigdaXaaacqGGUaGlaaGaaCzcaiaaxMaadaqadaqaaiabiodaZaGaayjkaiaawMcaaaaa@D4B2@

The stoichiometric coefficients *α*_*i *_and *β*_*j *_appear as exponents in the numerator and determine the orders of the polynomials in the denominator.

Reaction velocities do not only depend on reactant concentrations, but can also be controlled by modifiers. For each of them, we multiply eqn. (3) by a prefactor

hA(d,kA)=dkA+dor alternatively,hA(d,kA)=1+dkA     (4)
 MathType@MTEF@5@5@+=feaafiart1ev1aaatCvAUfKttLearuWrP9MDH5MBPbIqV92AaeXatLxBI9gBaebbnrfifHhDYfgasaacH8akY=wiFfYdH8Gipec8Eeeu0xXdbba9frFj0=OqFfea0dXdd9vqai=hGuQ8kuc9pgc9s8qqaq=dirpe0xb9q8qiLsFr0=vr0=vr0dc8meaabaqaciaacaGaaeqabaqabeGadaaakeaafaqadeGaeaaaaeaaaeaacqWGObaAdaWgaaWcbaacbaGae8xqaeeabeaakiabcIcaOiabdsgaKjabcYcaSiabdUgaRnaaCaaaleqabaGae8xqaeeaaOGaeiykaKcabaGaeyypa0dabaWaaSaaaeaacqWGKbazaeaacqWGRbWAdaahaaWcbeqaaiab=feabbaakiabgUcaRiabdsgaKbaaaeaacqWFVbWBcqWFYbGCcqqGGaaicqqGHbqycqqGSbaBcqqG0baDcqqGLbqzcqqGYbGCcqqGUbGBcqqGHbqycqqG0baDcqqGPbqAcqqG2bGDcqqGLbqzcqqGSbaBcqqG5bqEcqqGSaalaeaacqWGObaAdaWgaaWcbaGae8xqaeeabeaakiabcIcaOiabdsgaKjabcYcaSiabdUgaRnaaCaaaleqabaGae8xqaeeaaOGaeiykaKcabaGaeyypa0dabaGaeGymaeJaey4kaSYaaSaaaeaacqWGKbazaeaacqWGRbWAdaahaaWcbeqaaiab=feabbaaaaaaaOGaaCzcaiaaxMaadaqadaqaaiabisda0aGaayjkaiaawMcaaaaa@671E@

for an activator and

hI(d,kI)=kIkI+d     (5)
 MathType@MTEF@5@5@+=feaafiart1ev1aaatCvAUfKttLearuWrP9MDH5MBPbIqV92AaeXatLxBI9gBaebbnrfifHhDYfgasaacH8akY=wiFfYdH8Gipec8Eeeu0xXdbba9frFj0=OqFfea0dXdd9vqai=hGuQ8kuc9pgc9s8qqaq=dirpe0xb9q8qiLsFr0=vr0=vr0dc8meaabaqaciaacaGaaeqabaqabeGadaaakeaacqWGObaAdaWgaaWcbaacbaGae8xsaKeabeaakiabcIcaOiabdsgaKjabcYcaSiabdUgaRnaaCaaaleqabaGae8xsaKeaaOGaeiykaKIaeyypa0ZaaSaaaeaacqWGRbWAdaahaaWcbeqaaiab=LeajbaaaOqaaiabdUgaRnaaCaaaleqabaGae8xsaKeaaOGaey4kaSIaemizaqgaaiaaxMaacaWLjaWaaeWaaeaacqaI1aqnaiaawIcacaGLPaaaaaa@4253@

for an inhibitor. The activation constants *k*^A ^and inhibition constants *k*^I ^are measured in mM, and *d *is the concentration of the modifier.

### Convenience kinetics represents a random-order enzyme mechanism

Like many established rate laws (first of all, irreversible Michaelis-Menten kinetics [20]), convenience kinetics can be derived from a molecular enzyme mechanism. We impose three main assumptions: (i) the substrates bind to the enzyme in arbitrary order and are converted into the products, which then dissociate from the enzyme in arbitrary order; (ii) binding of substrates and products is reversible and much faster than the conversion step; (iii) the binding energies of individual reactants do not depend on other reactants already bound to the enzyme.

We shall demonstrate how the convenience rate law is derived for a bimolecular reaction

*A *+ *X *↔ *B *+ *Y*

without enzyme regulation. The reaction mechanism looks as follows:



The letters A, X, B, Y denote the reactants, *E*_0 _is the free enzyme, and *E*_A_, *E*_X_, *E*_AX_, *E*_B_, *E*_Y_, and *E*_BY _denote complexes of the enzyme and different combinations of reactants. We shall denote their concentrations by brackets (e.g., [*E*_A_]), the total enzyme concentration by *E*, and the concentrations of small metabolites by small letters (e.g., *a *= [A]).

The reaction proceeds from left to right; the free enzyme *E*_0 _binds to the substrates A and X in arbitrary order, forming the complexes *E*_A_, *E*_X_, and *E*_AX_. The binding of A can be described by an energy, the standard Gibbs free energy ΔGA(0)=GE(0)+GA(0)−GEA(0)
 MathType@MTEF@5@5@+=feaafiart1ev1aaatCvAUfKttLearuWrP9MDH5MBPbIqV92AaeXatLxBI9gBaebbnrfifHhDYfgasaacH8akY=wiFfYdH8Gipec8Eeeu0xXdbba9frFj0=OqFfea0dXdd9vqai=hGuQ8kuc9pgc9s8qqaq=dirpe0xb9q8qiLsFr0=vr0=vr0dc8meaabaqaciaacaGaaeqabaqabeGadaaakeaacqqHuoarcqWGhbWrdaqhaaWcbaacbaGae8xqaeeabaGaeiikaGIaeGimaaJaeiykaKcaaOGaeyypa0Jaem4raC0aa0baaSqaaiab=veafbqaaiabcIcaOiabicdaWiabcMcaPaaakiabgUcaRiabdEeahnaaDaaaleaacqWFbbqqaeaacqGGOaakcqaIWaamcqGGPaqkaaGccqGHsislcqWGhbWrdaqhaaWcbaGae8xrau0aaSbaaWqaaiab=feabbqabaaaleaacqGGOaakcqaIWaamcqGGPaqkaaaaaa@4609@ that is necessary to detach A from the complex *E*_A_. The dissociation constant kAM
 MathType@MTEF@5@5@+=feaafiart1ev1aaatCvAUfKttLearuWrP9MDH5MBPbIqV92AaeXatLxBI9gBaebbnrfifHhDYfgasaacH8akY=wiFfYdH8Gipec8Eeeu0xXdbba9frFj0=OqFfea0dXdd9vqai=hGuQ8kuc9pgc9s8qqaq=dirpe0xb9q8qiLsFr0=vr0=vr0dc8meaabaqaciaacaGaaeqabaqabeGadaaakeaacqWGRbWAdaqhaaWcbaacbaGae8xqaeeabaGae8xta0eaaaaa@3067@ = (*a *[*E*_0_])/[*E*_A_] describes the balance of bound and unbound A in chemical equilibrium and can be computed from the Gibbs free energy (in kJ/mol)

kAM=e−ΔGA(0)/RTmM     (6)
 MathType@MTEF@5@5@+=feaafiart1ev1aaatCvAUfKttLearuWrP9MDH5MBPbIqV92AaeXatLxBI9gBaebbnrfifHhDYfgasaacH8akY=wiFfYdH8Gipec8Eeeu0xXdbba9frFj0=OqFfea0dXdd9vqai=hGuQ8kuc9pgc9s8qqaq=dirpe0xb9q8qiLsFr0=vr0=vr0dc8meaabaqaciaacaGaaeqabaqabeGadaaakeaacqWGRbWAdaqhaaWcbaacbaGae8xqaeeabaGae8xta0eaaOGaeyypa0Jae8xzau2aaWbaaSqabeaacqGHsislcqqHuoarcqWGhbWrdaqhaaadbaGae8xqaeeabaGaeiikaGIaeGimaaJaeiykaKcaaSGaei4la8IaemOuaiLaemivaqfaaOGae8xBa0Mae8xta0KaaCzcaiaaxMaadaqadaqaaiabiAda2aGaayjkaiaawMcaaaaa@43D0@

with *RT *≈ 2.490 kJ/mol.

We now make a simplifying assumption: the binding energy of A does not depend on whether X is already bound. With analogous assumptions for binding of X and with the abbreviations a˜=a/kAM
 MathType@MTEF@5@5@+=feaafiart1ev1aaatCvAUfKttLearuWrP9MDH5MBPbIqV92AaeXatLxBI9gBaebbnrfifHhDYfgasaacH8akY=wiFfYdH8Gipec8Eeeu0xXdbba9frFj0=OqFfea0dXdd9vqai=hGuQ8kuc9pgc9s8qqaq=dirpe0xb9q8qiLsFr0=vr0=vr0dc8meaabaqaciaacaGaaeqabaqabeGadaaakeaacuWGHbqygaacaiabg2da9iabdggaHjabc+caViabdUgaRnaaDaaaleaacqqGbbqqaeaacqqGnbqtaaaaaa@34F3@, x˜=x/kXM
 MathType@MTEF@5@5@+=feaafiart1ev1aaatCvAUfKttLearuWrP9MDH5MBPbIqV92AaeXatLxBI9gBaebbnrfifHhDYfgasaacH8akY=wiFfYdH8Gipec8Eeeu0xXdbba9frFj0=OqFfea0dXdd9vqai=hGuQ8kuc9pgc9s8qqaq=dirpe0xb9q8qiLsFr0=vr0=vr0dc8meaabaqaciaacaGaaeqabaqabeGadaaakeaacuWG4baEgaacaiabg2da9iabdIha4jabc+caViabdUgaRnaaDaaaleaacqqGybawaeaacqqGnbqtaaaaaa@357D@, the equilibrium concentrations of the substrate complexes can be written as [*E*_A_] = *ã *[*E*_0_], [*E*_X_] = x˜
 MathType@MTEF@5@5@+=feaafiart1ev1aaatCvAUfKttLearuWrP9MDH5MBPbIqV92AaeXatLxBI9gBaebbnrfifHhDYfgasaacH8akY=wiFfYdH8Gipec8Eeeu0xXdbba9frFj0=OqFfea0dXdd9vqai=hGuQ8kuc9pgc9s8qqaq=dirpe0xb9q8qiLsFr0=vr0=vr0dc8meaabaqaciaacaGaaeqabaqabeGadaaakeaacuWG4baEgaacaaaa@2E34@[*E*_0_], [*E*_AX_] = *ã*x˜
 MathType@MTEF@5@5@+=feaafiart1ev1aaatCvAUfKttLearuWrP9MDH5MBPbIqV92AaeXatLxBI9gBaebbnrfifHhDYfgasaacH8akY=wiFfYdH8Gipec8Eeeu0xXdbba9frFj0=OqFfea0dXdd9vqai=hGuQ8kuc9pgc9s8qqaq=dirpe0xb9q8qiLsFr0=vr0=vr0dc8meaabaqaciaacaGaaeqabaqabeGadaaakeaacuWG4baEgaacaaaa@2E34@[*E*_0_]. By analogy, we obtain expressions for the product complexes on the right hand side: [*E*_B_] = b˜
 MathType@MTEF@5@5@+=feaafiart1ev1aaatCvAUfKttLearuWrP9MDH5MBPbIqV92AaeXatLxBI9gBaebbnrfifHhDYfgasaacH8akY=wiFfYdH8Gipec8Eeeu0xXdbba9frFj0=OqFfea0dXdd9vqai=hGuQ8kuc9pgc9s8qqaq=dirpe0xb9q8qiLsFr0=vr0=vr0dc8meaabaqaciaacaGaaeqabaqabeGadaaakeaacuWGIbGygaacaaaa@2E08@[*E*_0_], [*E*_Y_] = y˜
 MathType@MTEF@5@5@+=feaafiart1ev1aaatCvAUfKttLearuWrP9MDH5MBPbIqV92AaeXatLxBI9gBaebbnrfifHhDYfgasaacH8akY=wiFfYdH8Gipec8Eeeu0xXdbba9frFj0=OqFfea0dXdd9vqai=hGuQ8kuc9pgc9s8qqaq=dirpe0xb9q8qiLsFr0=vr0=vr0dc8meaabaqaciaacaGaaeqabaqabeGadaaakeaacuWG5bqEgaacaaaa@2E36@[*E*_0_], [*E*_BY_] = b˜
 MathType@MTEF@5@5@+=feaafiart1ev1aaatCvAUfKttLearuWrP9MDH5MBPbIqV92AaeXatLxBI9gBaebbnrfifHhDYfgasaacH8akY=wiFfYdH8Gipec8Eeeu0xXdbba9frFj0=OqFfea0dXdd9vqai=hGuQ8kuc9pgc9s8qqaq=dirpe0xb9q8qiLsFr0=vr0=vr0dc8meaabaqaciaacaGaaeqabaqabeGadaaakeaacuWGIbGygaacaaaa@2E08@y˜
 MathType@MTEF@5@5@+=feaafiart1ev1aaatCvAUfKttLearuWrP9MDH5MBPbIqV92AaeXatLxBI9gBaebbnrfifHhDYfgasaacH8akY=wiFfYdH8Gipec8Eeeu0xXdbba9frFj0=OqFfea0dXdd9vqai=hGuQ8kuc9pgc9s8qqaq=dirpe0xb9q8qiLsFr0=vr0=vr0dc8meaabaqaciaacaGaaeqabaqabeGadaaakeaacuWG5bqEgaacaaaa@2E36@[*E*_0_]. The total enzyme concentration *E *is the sum over the concentrations of all enzyme complexes

E=[E0] (1+a˜+x˜+a˜x˜+b˜+y˜+b˜y˜).     (7)
 MathType@MTEF@5@5@+=feaafiart1ev1aaatCvAUfKttLearuWrP9MDH5MBPbIqV92AaeXatLxBI9gBaebbnrfifHhDYfgasaacH8akY=wiFfYdH8Gipec8Eeeu0xXdbba9frFj0=OqFfea0dXdd9vqai=hGuQ8kuc9pgc9s8qqaq=dirpe0xb9q8qiLsFr0=vr0=vr0dc8meaabaqaciaacaGaaeqabaqabeGadaaakeaacqWGfbqrcqGH9aqpcqGGBbWwcqWGfbqrdaWgaaWcbaGaeGimaadabeaakiabc2faDjabbccaGiabcIcaOiabigdaXiabgUcaRiqbdggaHzaaiaGaey4kaSIafmiEaGNbaGaacqGHRaWkcuWGHbqygaacaiqbdIha4zaaiaGaey4kaSIafmOyaiMbaGaacqGHRaWkcuWG5bqEgaacaiabgUcaRiqbdkgaIzaaiaGafmyEaKNbaGaacqGGPaqkcqGGUaGlcaWLjaGaaCzcamaabmaabaGaeG4naCdacaGLOaGaayzkaaaaaa@4C6E@

We next assume a reversible conversion between the complexes *E*_AX _and *E*_BY _with forward and backward rate constants k+cat
 MathType@MTEF@5@5@+=feaafiart1ev1aaatCvAUfKttLearuWrP9MDH5MBPbIqV92AaeXatLxBI9gBaebbnrfifHhDYfgasaacH8akY=wiFfYdH8Gipec8Eeeu0xXdbba9frFj0=OqFfea0dXdd9vqai=hGuQ8kuc9pgc9s8qqaq=dirpe0xb9q8qiLsFr0=vr0=vr0dc8meaabaqaciaacaGaaeqabaqabeGadaaakeaacqWGRbWAdaqhaaWcbaGaey4kaScabaacbaGae83yamMae8xyaeMae8hDaqhaaaaa@3322@ and k−cat
 MathType@MTEF@5@5@+=feaafiart1ev1aaatCvAUfKttLearuWrP9MDH5MBPbIqV92AaeXatLxBI9gBaebbnrfifHhDYfgasaacH8akY=wiFfYdH8Gipec8Eeeu0xXdbba9frFj0=OqFfea0dXdd9vqai=hGuQ8kuc9pgc9s8qqaq=dirpe0xb9q8qiLsFr0=vr0=vr0dc8meaabaqaciaacaGaaeqabaqabeGadaaakeaacqWGRbWAdaqhaaWcbaGaeyOeI0cabaacbaGae83yamMae8xyaeMae8hDaqhaaaaa@332D@; this reaction step determines the overall reaction rate. Its velocity reads

v(a,x,b,y)=k+cat[EAX]−k−cat[EBY]=k+cata˜x˜[E0]−k−catb˜y˜[E0]=Ek+cata˜x˜−k−catb˜y˜1+a˜+x˜+a˜x˜+b˜+y˜+b˜y˜=Ek+cata˜x˜−k−catb˜y˜(1+a˜)(1+x˜)+(1+b˜)(1+y˜)−1,     (8)
 MathType@MTEF@5@5@+=feaafiart1ev1aaatCvAUfKttLearuWrP9MDH5MBPbIqV92AaeXatLxBI9gBaebbnrfifHhDYfgasaacH8akY=wiFfYdH8Gipec8Eeeu0xXdbba9frFj0=OqFfea0dXdd9vqai=hGuQ8kuc9pgc9s8qqaq=dirpe0xb9q8qiLsFr0=vr0=vr0dc8meaabaqaciaacaGaaeqabaqabeGadaaakeaafaqaaeabdaaaaeaacqWG2bGDcqGGOaakcqWGHbqycqGGSaalcqWG4baEcqGGSaalcqWGIbGycqGGSaalcqWG5bqEcqGGPaqkaeaacqGH9aqpaeaacqWGRbWAdaqhaaWcbaGaey4kaScabaGaee4yamMaeeyyaeMaeeiDaqhaaOGaei4waSLaemyrau0aaSbaaSqaaiabbgeabjabbIfaybqabaGccqGGDbqxcqGHsislcqWGRbWAdaqhaaWcbaGaeyOeI0cabaGaee4yamMaeeyyaeMaeeiDaqhaaOGaei4waSLaemyrau0aaSbaaSqaaiabbkeacjabbMfazbqabaGccqGGDbqxaeaaaeaacqGH9aqpaeaacqWGRbWAdaqhaaWcbaGaey4kaScabaGaee4yamMaeeyyaeMaeeiDaqhaaOGafmyyaeMbaGaacuWG4baEgaacaiabcUfaBjabdweafnaaBaaaleaacqaIWaamaeqaaOGaeiyxa0LaeyOeI0Iaem4AaS2aa0baaSqaaiabgkHiTaqaaiabbogaJjabbggaHjabbsha0baakiqbdkgaIzaaiaGafmyEaKNbaGaacqGGBbWwcqWGfbqrdaWgaaWcbaGaeGimaadabeaakiabc2faDbqaaaqaaiabg2da9aqaaiabdweafnaalaaabaGaem4AaS2aa0baaSqaaiabgUcaRaqaaiabbogaJjabbggaHjabbsha0baakiqbdggaHzaaiaGafmiEaGNbaGaacqGHsislcqWGRbWAdaqhaaWcbaGaeyOeI0cabaGaee4yamMaeeyyaeMaeeiDaqhaaOGafmOyaiMbaGaacuWG5bqEgaacaaqaaiabigdaXiabgUcaRiqbdggaHzaaiaGaey4kaSIafmiEaGNbaGaacqGHRaWkcuWGHbqygaacaiqbdIha4zaaiaGaey4kaSIafmOyaiMbaGaacqGHRaWkcuWG5bqEgaacaiabgUcaRiqbdkgaIzaaiaGafmyEaKNbaGaaaaaabaaabaGaeyypa0dabaGaemyrau0aaSaaaeaacqWGRbWAdaqhaaWcbaGaey4kaScabaGaee4yamMaeeyyaeMaeeiDaqhaaOGafmyyaeMbaGaacuWG4baEgaacaiabgkHiTiabdUgaRnaaDaaaleaacqGHsislaeaacqqGJbWycqqGHbqycqqG0baDaaGccuWGIbGygaacaiqbdMha5zaaiaaabaGaeiikaGIaeGymaeJaey4kaSIafmyyaeMbaGaacqGGPaqkcqGGOaakcqaIXaqmcqGHRaWkcuWG4baEgaacaiabcMcaPiabgUcaRiabcIcaOiabigdaXiabgUcaRiqbdkgaIzaaiaGaeiykaKIaeiikaGIaeGymaeJaey4kaSIafmyEaKNbaGaacqGGPaqkcqGHsislcqaIXaqmaaGaeiilaWIaaCzcaiaaxMaadaqadaqaaiabiIda4aGaayjkaiaawMcaaaaaaaa@CA29@

which is exactly the convenience rate law (2). The derivation has shown that the turnover rates k±cat
 MathType@MTEF@5@5@+=feaafiart1ev1aaatCvAUfKttLearuWrP9MDH5MBPbIqV92AaeXatLxBI9gBaebbnrfifHhDYfgasaacH8akY=wiFfYdH8Gipec8Eeeu0xXdbba9frFj0=OqFfea0dXdd9vqai=hGuQ8kuc9pgc9s8qqaq=dirpe0xb9q8qiLsFr0=vr0=vr0dc8meaabaqaciaacaGaaeqabaqabeGadaaakeaacqWGRbWAdaqhaaWcbaGaeyySaelabaacbaGae83yamMae8xyaeMae8hDaqhaaaaa@342E@ stem from the conversion step, while the reactant constants *k*^M ^are actually dissociation constants, related to the binding energies between reactants and enzyme. The terms in the denominator represent the enzyme complexes in the reaction scheme shown above. Equation (8) also shows why the term -1 in formulae (2) and (3) is necessary: the two product terms in the denominator represent all complexes shown in the reaction scheme. However, when summing up the terms from both sides, we counted the free enzyme *E*_0 _twice, so we have to subtract it once.

The same kind of argument can be applied to reactions with other stoichiometries; let us consider a reaction with the left-hand side 2 A + X ↔ ...



The substrate complex *E*_AAX _gives rise to the first term k+cat
 MathType@MTEF@5@5@+=feaafiart1ev1aaatCvAUfKttLearuWrP9MDH5MBPbIqV92AaeXatLxBI9gBaebbnrfifHhDYfgasaacH8akY=wiFfYdH8Gipec8Eeeu0xXdbba9frFj0=OqFfea0dXdd9vqai=hGuQ8kuc9pgc9s8qqaq=dirpe0xb9q8qiLsFr0=vr0=vr0dc8meaabaqaciaacaGaaeqabaqabeGadaaakeaacqWGRbWAdaqhaaWcbaGaey4kaScabaacbaGae83yamMae8xyaeMae8hDaqhaaaaa@3322@*ã*^2^x˜
 MathType@MTEF@5@5@+=feaafiart1ev1aaatCvAUfKttLearuWrP9MDH5MBPbIqV92AaeXatLxBI9gBaebbnrfifHhDYfgasaacH8akY=wiFfYdH8Gipec8Eeeu0xXdbba9frFj0=OqFfea0dXdd9vqai=hGuQ8kuc9pgc9s8qqaq=dirpe0xb9q8qiLsFr0=vr0=vr0dc8meaabaqaciaacaGaaeqabaqabeGadaaakeaacuWG4baEgaacaaaa@2E34@ in the numerator, with the stoichiometric coefficient in the exponent. In the denominator, each term corresponds to one of the enzyme complexes, yielding

1+a˜+x˜+a˜x˜+a˜2+a˜2x˜+...=(1+a˜+a˜2)(1+x˜)+...     (9)
 MathType@MTEF@5@5@+=feaafiart1ev1aaatCvAUfKttLearuWrP9MDH5MBPbIqV92AaeXatLxBI9gBaebbnrfifHhDYfgasaacH8akY=wiFfYdH8Gipec8Eeeu0xXdbba9frFj0=OqFfea0dXdd9vqai=hGuQ8kuc9pgc9s8qqaq=dirpe0xb9q8qiLsFr0=vr0=vr0dc8meaabaqaciaacaGaaeqabaqabeGadaaakeaacqaIXaqmcqGHRaWkcuWGHbqygaacaiabgUcaRiqbdIha4zaaiaGaey4kaSIafmyyaeMbaGaacuWG4baEgaacaiabgUcaRiqbdggaHzaaiaWaaWbaaSqabeaacqaIYaGmaaGccqGHRaWkcuWGHbqygaacamaaCaaaleqabaGaeGOmaidaaOGafmiEaGNbaGaacqGHRaWkcqGGUaGlcqGGUaGlcqGGUaGlcqGH9aqpcqGGOaakcqaIXaqmcqGHRaWkcuWGHbqygaacaiabgUcaRiqbdggaHzaaiaWaaWbaaSqabeaacqaIYaGmaaGccqGGPaqkcqGGOaakcqaIXaqmcqGHRaWkcuWG4baEgaacaiabcMcaPiabgUcaRiabc6caUiabc6caUiabc6caUiaaxMaacaWLjaWaaeWaaeaacqaI5aqoaiaawIcacaGLPaaaaaa@5796@

where the dots still denote the terms from the right-hand side. The shape of the two factors, (1 + *ã *+ *ã*^2^) and (1 + x˜
 MathType@MTEF@5@5@+=feaafiart1ev1aaatCvAUfKttLearuWrP9MDH5MBPbIqV92AaeXatLxBI9gBaebbnrfifHhDYfgasaacH8akY=wiFfYdH8Gipec8Eeeu0xXdbba9frFj0=OqFfea0dXdd9vqai=hGuQ8kuc9pgc9s8qqaq=dirpe0xb9q8qiLsFr0=vr0=vr0dc8meaabaqaciaacaGaaeqabaqabeGadaaakeaacuWG4baEgaacaaaa@2E34@), corresponds to the rows and columns in the above scheme.

The activation and inhibition terms in the prefactor can also be justified mechanistically: in addition to binding sites for reactants, the enzyme contains binding sites for activators and inhibitors. Only those enzyme molecules to which all activators and none of the inhibitors are bound contribute to the reaction mechanism; all other enzyme molecules are inactive. Again, we assume that the Gibbs free energies for binding do not depend on whether other modifiers are bound, and they determine the *k*^A ^and *k*^I ^values as in eqn. (6).

To define a convenience kinetics for irreversible reactions, we assume that all product constants kbM
 MathType@MTEF@5@5@+=feaafiart1ev1aaatCvAUfKttLearuWrP9MDH5MBPbIqV92AaeXatLxBI9gBaebbnrfifHhDYfgasaacH8akY=wiFfYdH8Gipec8Eeeu0xXdbba9frFj0=OqFfea0dXdd9vqai=hGuQ8kuc9pgc9s8qqaq=dirpe0xb9q8qiLsFr0=vr0=vr0dc8meaabaqaciaacaGaaeqabaqabeGadaaakeaacqWGRbWAdaqhaaWcbaacbaGae8NyaigabaGae8xta0eaaaaa@30A9@ – and thereby the overall equilibrium constant, as will be explained below – go to infinity. In the enzymatic mechanism, binding between products and enzyme becomes energetically very unfavourable. As a consequence, all b˜
 MathType@MTEF@5@5@+=feaafiart1ev1aaatCvAUfKttLearuWrP9MDH5MBPbIqV92AaeXatLxBI9gBaebbnrfifHhDYfgasaacH8akY=wiFfYdH8Gipec8Eeeu0xXdbba9frFj0=OqFfea0dXdd9vqai=hGuQ8kuc9pgc9s8qqaq=dirpe0xb9q8qiLsFr0=vr0=vr0dc8meaabaqaciaacaGaaeqabaqabeGadaaakeaacuWGIbGygaacaaaa@2E08@_*j *_in eqn. (3) vanish and we obtain the irreversible rate law

v(a)=E k+cat∏ia˜iαi∑m=0αi(a˜i)m=E k+cat∏i(∑m=0αi(a˜i)−m)−1.     (10)
 MathType@MTEF@5@5@+=feaafiart1ev1aaatCvAUfKttLearuWrP9MDH5MBPbIqV92AaeXatLxBI9gBaebbnrfifHhDYfgasaacH8akY=wiFfYdH8Gipec8Eeeu0xXdbba9frFj0=OqFfea0dXdd9vqai=hGuQ8kuc9pgc9s8qqaq=dirpe0xb9q8qiLsFr0=vr0=vr0dc8meaabaqaciaacaGaaeqabaqabeGadaaakeaacqWG2bGDcqGGOaakieqacqWFHbqycqGGPaqkcqGH9aqpcqWGfbqrcqqGGaaiieGacqGFRbWAdaqhaaWcbaGae43kaScabaacbaGae03yamMae0xyaeMae0hDaqhaaOWaaebuaeaadaWcaaqaaiqbdggaHzaaiaWaa0baaSqaaiabdMgaPbqaaGGaciab8f7aHnaaBaaameaacqWGPbqAaeqaaaaaaOqaamaaqahabaGaeiikaGIafmyyaeMbaGaadaWgaaWcbaGaemyAaKgabeaakiabcMcaPmaaCaaaleqabaGaemyBa0gaaaqaaiabd2gaTjabg2da9iabicdaWaqaaiab8f7aHnaaBaaameaacqWGPbqAaeqaaaqdcqGHris5aaaakiabg2da9iabdweafjabbccaGiab+TgaRnaaDaaaleaacqGFRaWkaeaacqqFJbWycqqFHbqycqqF0baDaaGcdaqeqbqaamaabmaabaWaaabCaeaacqGGOaakcuWGHbqygaacamaaBaaaleaacqWGPbqAaeqaaOGaeiykaKYaaWbaaSqabeaacqGHsislcqWGTbqBaaaabaGaemyBa0Maeyypa0JaeGimaadabaGaeWxSde2aaSbaaWqaaiabdMgaPbqabaaaniabggHiLdaakiaawIcacaGLPaaaaSqaaiab+LgaPbqab0Gaey4dIunakmaaCaaaleqabaGaeyOeI0IaeGymaedaaaqaaiabdMgaPbqab0Gaey4dIunakiabc6caUiaaxMaacaWLjaWaaeWaaeaacqaIXaqmcqaIWaamaiaawIcacaGLPaaaaaa@789C@

### The reactant constants denote half-saturation concentrations

Besides being a dissociation constant, the *k*^M ^value in Michaelis-Menten kinetics (1) has a simple mathematical meaning: it denotes the substrate concentration that leads to a half-maximal reaction velocity if the product is absent. A similar rule holds for the substrate and product constants in convenience kinetics. Let us first assume that all stoichiometric coefficients are ±1; if the product concentrations vanish (*b*_*j *_= 0), then rate law (2) can be factorised into

v(a,0)=E k+cat∏ia˜i1+a˜i.     (11)
 MathType@MTEF@5@5@+=feaafiart1ev1aaatCvAUfKttLearuWrP9MDH5MBPbIqV92AaeXatLxBI9gBaebbnrfifHhDYfgasaacH8akY=wiFfYdH8Gipec8Eeeu0xXdbba9frFj0=OqFfea0dXdd9vqai=hGuQ8kuc9pgc9s8qqaq=dirpe0xb9q8qiLsFr0=vr0=vr0dc8meaabaqaciaacaGaaeqabaqabeGadaaakeaacqWG2bGDcqGGOaakieqacqWFHbqycqGGSaalcqaIWaamcqGGPaqkcqGH9aqpcqWGfbqrcqqGGaaiieGacqGFRbWAdaqhaaWcbaGae43kaScabaacbaGae03yamMae0xyaeMae0hDaqhaaOWaaebuaeaadaWcaaqaaiqbdggaHzaaiaWaaSbaaSqaaiabdMgaPbqabaaakeaacqaIXaqmcqGHRaWkcuWGHbqygaacamaaBaaaleaacqWGPbqAaeqaaaaaaeaacqWGPbqAaeqaniabg+GivdGccqGGUaGlcaWLjaGaaCzcamaabmaabaGaeGymaeJaeGymaedacaGLOaGaayzkaaaaaa@4CF5@

If in addition, all substrate concentrations except for a certain *a*_*m *_are kept fixed, the rate law reads

v(a,0)=a˜m1+a˜m⋅const.     (12)
 MathType@MTEF@5@5@+=feaafiart1ev1aaatCvAUfKttLearuWrP9MDH5MBPbIqV92AaeXatLxBI9gBaebbnrfifHhDYfgasaacH8akY=wiFfYdH8Gipec8Eeeu0xXdbba9frFj0=OqFfea0dXdd9vqai=hGuQ8kuc9pgc9s8qqaq=dirpe0xb9q8qiLsFr0=vr0=vr0dc8meaabaqaciaacaGaaeqabaqabeGadaaakeaacqWG2bGDcqGGOaakieqacqWFHbqycqGGSaalcqaIWaamcqGGPaqkcqGH9aqpdaWcaaqaaiqbdggaHzaaiaWaaSbaaSqaaiabd2gaTbqabaaakeaacqaIXaqmcqGHRaWkcuWGHbqygaacamaaBaaaleaacqWGTbqBaeqaaaaakiabgwSixJqaaiab+ngaJjab+9gaVjab+5gaUjab+nhaZjab+rha0jabc6caUiaaxMaacaWLjaWaaeWaaeaacqaIXaqmcqaIYaGmaiaawIcacaGLPaaaaaa@4A88@

For *a*_*m *_→ ∞, the fraction approaches 1, while for *a*_*m *_= kaM
 MathType@MTEF@5@5@+=feaafiart1ev1aaatCvAUfKttLearuWrP9MDH5MBPbIqV92AaeXatLxBI9gBaebbnrfifHhDYfgasaacH8akY=wiFfYdH8Gipec8Eeeu0xXdbba9frFj0=OqFfea0dXdd9vqai=hGuQ8kuc9pgc9s8qqaq=dirpe0xb9q8qiLsFr0=vr0=vr0dc8meaabaqaciaacaGaaeqabaqabeGadaaakeaacqWGRbWAdaqhaaWcbaGaemyyaegabaacbaGae8xta0eaaaaa@30AB@ it yields 1/2. In particular, if all other substrates are present in high amounts, we obtain the half-maximal velocity, just as in Michaelis-Menten kinetics.

What if the stoichiometric coefficient is larger than one? Applying the same argument for *α*_*m *_= 2, we obtain the velocity

v(a,0)=a˜m21+a˜m+a˜m2⋅const.     (13)
 MathType@MTEF@5@5@+=feaafiart1ev1aaatCvAUfKttLearuWrP9MDH5MBPbIqV92AaeXatLxBI9gBaebbnrfifHhDYfgasaacH8akY=wiFfYdH8Gipec8Eeeu0xXdbba9frFj0=OqFfea0dXdd9vqai=hGuQ8kuc9pgc9s8qqaq=dirpe0xb9q8qiLsFr0=vr0=vr0dc8meaabaqaciaacaGaaeqabaqabeGadaaakeaacqWG2bGDcqGGOaakieqacqWFHbqycqGGSaalcqaIWaamcqGGPaqkcqGH9aqpdaWcaaqaaiqbdggaHzaaiaWaa0baaSqaaiabd2gaTbqaaiabikdaYaaaaOqaaiabigdaXiabgUcaRiqbdggaHzaaiaWaaSbaaSqaaiabd2gaTbqabaGccqGHRaWkcuWGHbqygaacamaaDaaaleaacqWGTbqBaeaacqaIYaGmaaaaaOGaeyyXICncbaGae43yamMae43Ba8Mae4NBa4Mae43CamNae4hDaqNaeiOla4IaaCzcaiaaxMaadaqadaqaaiabigdaXiabiodaZaGaayjkaiaawMcaaaaa@5045@

At *a*_*m *_= kaM
 MathType@MTEF@5@5@+=feaafiart1ev1aaatCvAUfKttLearuWrP9MDH5MBPbIqV92AaeXatLxBI9gBaebbnrfifHhDYfgasaacH8akY=wiFfYdH8Gipec8Eeeu0xXdbba9frFj0=OqFfea0dXdd9vqai=hGuQ8kuc9pgc9s8qqaq=dirpe0xb9q8qiLsFr0=vr0=vr0dc8meaabaqaciaacaGaaeqabaqabeGadaaakeaacqWGRbWAdaqhaaWcbaGaemyyaegabaacbaGae8xta0eaaaaa@30AB@, the ratio is 1/3, so the reaction rate is 1/3 of the maximal rate. Extending this argument to other stoichiometric coefficients *α*_*i*_, we can conclude: at *a*_*m *_= kaM
 MathType@MTEF@5@5@+=feaafiart1ev1aaatCvAUfKttLearuWrP9MDH5MBPbIqV92AaeXatLxBI9gBaebbnrfifHhDYfgasaacH8akY=wiFfYdH8Gipec8Eeeu0xXdbba9frFj0=OqFfea0dXdd9vqai=hGuQ8kuc9pgc9s8qqaq=dirpe0xb9q8qiLsFr0=vr0=vr0dc8meaabaqaciaacaGaaeqabaqabeGadaaakeaacqWGRbWAdaqhaaWcbaGaemyyaegabaacbaGae8xta0eaaaaa@30AB@, excess of all other substrates, and vanishing product concentrations, the reaction rate equals the maximal reaction rate divided by 1 + *α*_*i*_.

### Convenience kinetics for entire biochemical networks

To parametrise an entire metabolic network with stoichiometric matrix *N *and regulation matrix *W *(for notation, see methods section), it is practical to arrange the kinetic parameters in vectors and matrices. The enzyme concentration of a reaction *l *reads *E*_*l*_, and the turnover rates are called k±lcat
 MathType@MTEF@5@5@+=feaafiart1ev1aaatCvAUfKttLearuWrP9MDH5MBPbIqV92AaeXatLxBI9gBaebbnrfifHhDYfgasaacH8akY=wiFfYdH8Gipec8Eeeu0xXdbba9frFj0=OqFfea0dXdd9vqai=hGuQ8kuc9pgc9s8qqaq=dirpe0xb9q8qiLsFr0=vr0=vr0dc8meaabaqaciaacaGaaeqabaqabeGadaaakeaacqWGRbWAdaqhaaWcbaGaeyySaeRaemiBaWgabaacbaGae83yamMae8xyaeMae8hDaqhaaaaa@358F@. Each stoichiometric interaction (where *n*_*il *_≠ 0) comes with a value kliM
 MathType@MTEF@5@5@+=feaafiart1ev1aaatCvAUfKttLearuWrP9MDH5MBPbIqV92AaeXatLxBI9gBaebbnrfifHhDYfgasaacH8akY=wiFfYdH8Gipec8Eeeu0xXdbba9frFj0=OqFfea0dXdd9vqai=hGuQ8kuc9pgc9s8qqaq=dirpe0xb9q8qiLsFr0=vr0=vr0dc8meaabaqaciaacaGaaeqabaqabeGadaaakeaacqWGRbWAdaqhaaWcbaGaemiBaWMaemyAaKgabaacbaGae8xta0eaaaaa@321C@, while activation (*w*_*li *_= 1) and inhibition (*w*_*li *_= -1) are quantified by values kliA
 MathType@MTEF@5@5@+=feaafiart1ev1aaatCvAUfKttLearuWrP9MDH5MBPbIqV92AaeXatLxBI9gBaebbnrfifHhDYfgasaacH8akY=wiFfYdH8Gipec8Eeeu0xXdbba9frFj0=OqFfea0dXdd9vqai=hGuQ8kuc9pgc9s8qqaq=dirpe0xb9q8qiLsFr0=vr0=vr0dc8meaabaqaciaacaGaaeqabaqabeGadaaakeaacqWGRbWAdaqhaaWcbaGaemiBaWMaemyAaKgabaacbaGae8xqaeeaaaaa@3204@ and kliI
 MathType@MTEF@5@5@+=feaafiart1ev1aaatCvAUfKttLearuWrP9MDH5MBPbIqV92AaeXatLxBI9gBaebbnrfifHhDYfgasaacH8akY=wiFfYdH8Gipec8Eeeu0xXdbba9frFj0=OqFfea0dXdd9vqai=hGuQ8kuc9pgc9s8qqaq=dirpe0xb9q8qiLsFr0=vr0=vr0dc8meaabaqaciaacaGaaeqabaqabeGadaaakeaacqWGRbWAdaqhaaWcbaGaemiBaWMaemyAaKgabaacbaGae8xsaKeaaaaa@3214@, respectively. The *k*^M^, *k*^A ^and *k*^I ^values for non-existing interactions (where *n*_*il *_= 0 or *w*_*li *_= 0) remain unspecified or can be assigned a value of 1, i.e., a logarithmic value of 0.

With metabolite concentrations arranged in a vector **c**, the convenience kinetics can now be written as

vl=El∏mhA(cm,klmA)wlm+hI(cm,klmI)wlm−k+lcat∏ic˜linil−−k−lcat∏ic˜linil+∏i∑m=0nil−(c˜li)m+∏i∑m=0nil+(c˜li)m−1.     (14)
 MathType@MTEF@5@5@+=feaafiart1ev1aaatCvAUfKttLearuWrP9MDH5MBPbIqV92AaeXatLxBI9gBaebbnrfifHhDYfgasaacH8akY=wiFfYdH8Gipec8Eeeu0xXdbba9frFj0=OqFfea0dXdd9vqai=hGuQ8kuc9pgc9s8qqaq=dirpe0xb9q8qiLsFr0=vr0=vr0dc8meaabaqaciaacaGaaeqabaqabeGadaaakeaacqWG2bGDdaWgaaWcbaGaemiBaWgabeaakiabg2da9iabdweafnaaBaaaleaacqWGSbaBaeqaaOWaaebuaeaacqWGObaAdaWgaaWcbaGaeeyqaeeabeaaaeaacqWGTbqBaeqaniabg+GivdGccqGGOaakcqWGJbWydaWgaaWcbaGaemyBa0gabeaakiabcYcaSiabdUgaRnaaDaaaleaacqWGSbaBcqWGTbqBaeaacqqGbbqqaaGccqGGPaqkdaahaaWcbeqaaiabdEha3naaDaaameaacqWGSbaBcqWGTbqBaeaacqGHRaWkaaaaaOGaemiAaG2aaSbaaSqaaiabbMeajbqabaGccqGGOaakcqWGJbWydaWgaaWcbaGaemyBa0gabeaakiabcYcaSiabdUgaRnaaDaaaleaacqWGSbaBcqWGTbqBaeaacqqGjbqsaaGccqGGPaqkdaahaaWcbeqaaiabdEha3naaDaaameaacqWGSbaBcqWGTbqBaeaacqGHsislaaaaaOWaaSaaaeaacqWGRbWAdaqhaaWcbaGaey4kaSIaemiBaWgabaGaee4yamMaeeyyaeMaeeiDaqhaaOWaaebuaeaacuWGJbWygaacamaaDaaaleaacqWGSbaBcqWGPbqAaeaacqWGUbGBdaqhaaadbaGaemyAaKMaemiBaWgabaGaeyOeI0caaaaaaSqaaiabdMgaPbqab0Gaey4dIunakiabgkHiTiabdUgaRnaaDaaaleaacqGHsislcqWGSbaBaeaacqqGJbWycqqGHbqycqqG0baDaaGcdaqeqbqaaiqbdogaJzaaiaWaa0baaSqaaiabdYgaSjabdMgaPbqaaiabd6gaUnaaDaaameaacqWGPbqAcqWGSbaBaeaacqGHRaWkaaaaaaWcbaGaemyAaKgabeqdcqGHpis1aaGcbaWaaebuaeaadaaeWbqaaiabcIcaOiqbdogaJzaaiaWaaSbaaSqaaiabdYgaSjabdMgaPbqabaGccqGGPaqkdaahaaWcbeqaaiabd2gaTbaaaeaacqWGTbqBcqGH9aqpcqaIWaamaeaacqWGUbGBdaqhaaadbaGaemyAaKMaemiBaWgabaGaeyOeI0caaaqdcqGHris5aOGaey4kaSYaaebuaeaadaaeWbqaaiabcIcaOiqbdogaJzaaiaWaaSbaaSqaaiabdYgaSjabdMgaPbqabaGccqGGPaqkdaahaaWcbeqaaiabd2gaTbaaaeaacqWGTbqBcqGH9aqpcqaIWaamaeaacqWGUbGBdaqhaaadbaGaemyAaKMaemiBaWgabaGaey4kaScaaaqdcqGHris5aOGaeyOeI0IaeGymaedaleaacqWGPbqAaeqaniabg+GivdaaleaacqWGPbqAaeqaniabg+GivdaaaOGaeiOla4IaaCzcaiaaxMaadaqadaqaaiabigdaXiabisda0aGaayjkaiaawMcaaaaa@BB93@

with the abbreviation c˜li=ci/kliM
 MathType@MTEF@5@5@+=feaafiart1ev1aaatCvAUfKttLearuWrP9MDH5MBPbIqV92AaeXatLxBI9gBaebbnrfifHhDYfgasaacH8akY=wiFfYdH8Gipec8Eeeu0xXdbba9frFj0=OqFfea0dXdd9vqai=hGuQ8kuc9pgc9s8qqaq=dirpe0xb9q8qiLsFr0=vr0=vr0dc8meaabaqaciaacaGaaeqabaqabeGadaaakeaacuWGJbWygaacamaaBaaaleaacqWGSbaBcqWGPbqAaeqaaOGaeyypa0Jaem4yam2aaSbaaSqaaiabdMgaPbqabaGccqGGVaWlcqWGRbWAdaqhaaWcbaGaemiBaWMaemyAaKgabaacbaGae8xta0eaaaaa@3B38@. For ease of notation here, we defined the matrices *N*^+ ^= (nil+
 MathType@MTEF@5@5@+=feaafiart1ev1aaatCvAUfKttLearuWrP9MDH5MBPbIqV92AaeXatLxBI9gBaebbnrfifHhDYfgasaacH8akY=wiFfYdH8Gipec8Eeeu0xXdbba9frFj0=OqFfea0dXdd9vqai=hGuQ8kuc9pgc9s8qqaq=dirpe0xb9q8qiLsFr0=vr0=vr0dc8meaabaqaciaacaGaaeqabaqabeGadaaakeaacqWGUbGBdaqhaaWcbaGaemyAaKMaemiBaWgabaGaey4kaScaaaaa@31DC@), *N*^- ^= (nil−
 MathType@MTEF@5@5@+=feaafiart1ev1aaatCvAUfKttLearuWrP9MDH5MBPbIqV92AaeXatLxBI9gBaebbnrfifHhDYfgasaacH8akY=wiFfYdH8Gipec8Eeeu0xXdbba9frFj0=OqFfea0dXdd9vqai=hGuQ8kuc9pgc9s8qqaq=dirpe0xb9q8qiLsFr0=vr0=vr0dc8meaabaqaciaacaGaaeqabaqabeGadaaakeaacqWGUbGBdaqhaaWcbaGaemyAaKMaemiBaWgabaGaeyOeI0caaaaa@31E7@), which respectively contain the absolute values of all positive and negative elements of *N*. The matrices *W*^+ ^and *W*^- ^are derived from *W *in the same way.

Let us add some remarks, (i) It is common to describe some of the metabolite concentrations by fixed values rather than by a balance equation. In the present framework, these metabolites are included in the concentration vector **c **and in the structure matrices *N *or *W*. (ii) A reaction is always catalysed by a specific enzyme; we describe isoenzymes by distinct reactions. (iii) If the sign of a regulatory interaction is unknown, we may consider terms for both activation and inhibition. (iv) To describe indirect regulation, e.g. by transcriptional control, the production and degradation of enzymes has to be modelled explicitly by chemical reactions.

### Thermodynamic dependence between parameters

The convenience kinetics (14) has a major drawback: its parameters are constrained by the second law of thermodynamics. The equilibrium constant of reaction *l *is defined as

kleq=∏i(cieq)nil     (15)
 MathType@MTEF@5@5@+=feaafiart1ev1aaatCvAUfKttLearuWrP9MDH5MBPbIqV92AaeXatLxBI9gBaebbnrfifHhDYfgasaacH8akY=wiFfYdH8Gipec8Eeeu0xXdbba9frFj0=OqFfea0dXdd9vqai=hGuQ8kuc9pgc9s8qqaq=dirpe0xb9q8qiLsFr0=vr0=vr0dc8meaabaqaciaacaGaaeqabaqabeGadaaakeaacqWGRbWAdaqhaaWcbaGaemiBaWgabaacbaGae8xzauMae8xCaehaaOGaeyypa0ZaaebuaeaacqGGOaakcqWGJbWydaqhaaWcbaGaemyAaKgabaGae8xzauMae8xCaehaaOGaeiykaKYaaWbaaSqabeaacqWGUbGBdaWgaaadbaGaemyAaKMaemiBaWgabeaaaaaaleaacqWGPbqAaeqaniabg+GivdGccaWLjaGaaCzcamaabmaabaGaeGymaeJaeGynaudacaGLOaGaayzkaaaaaa@4758@

where **c**^eq ^is a vector of metabolite concentrations in a chemical equilibrium state. By setting eqn. (3) to zero, we obtain the Haldane relationship [16] for the convenience kinetics,

keq=∏jbjβj∏iaiαi=k+catk−cat∏j(kbjM)βj∏i(kaiM)αi.     (16)
 MathType@MTEF@5@5@+=feaafiart1ev1aaatCvAUfKttLearuWrP9MDH5MBPbIqV92AaeXatLxBI9gBaebbnrfifHhDYfgasaacH8akY=wiFfYdH8Gipec8Eeeu0xXdbba9frFj0=OqFfea0dXdd9vqai=hGuQ8kuc9pgc9s8qqaq=dirpe0xb9q8qiLsFr0=vr0=vr0dc8meaabaqaciaacaGaaeqabaqabeGadaaakeaacqWGRbWAdaahaaWcbeqaaGqaaiab=vgaLjab=fhaXbaakiabg2da9maalaaabaWaaebuaeaacqWGIbGydaqhaaWcbaGaemOAaOgabaacciGae4NSdi2aaSbaaWqaaiabdQgaQbqabaaaaaWcbaGaemOAaOgabeqdcqGHpis1aaGcbaWaaebuaeaacqWGHbqydaqhaaWcbaGaemyAaKgabaGae4xSde2aaSbaaWqaaiabdMgaPbqabaaaaaWcbaGaemyAaKgabeqdcqGHpis1aaaakiabg2da9maalaaabaGaem4AaS2aa0baaSqaaiabgUcaRaqaaiab=ngaJjab=fgaHjab=rha0baaaOqaaiabdUgaRnaaDaaaleaacqGHsislaeaacqWFJbWycqWFHbqycqWF0baDaaaaaOWaaSaaaeaadaqeqbqaaiabcIcaOiabdUgaRnaaDaaaleaacqWFIbGydaWgaaadbaGaemOAaOgabeaaaSqaaiab=1eanbaaaeaacqWGQbGAaeqaniabg+GivdGccqGGPaqkdaahaaWcbeqaaiab+j7aInaaBaaameaacqWGQbGAaeqaaaaaaOqaamaarafabaGaeiikaGIaem4AaS2aa0baaSqaaiab=fgaHnaaBaaameaacqWGPbqAaeqaaaWcbaGae8xta0eaaaqaaiabdMgaPbqab0Gaey4dIunakiabcMcaPmaaCaaaleqabaGae4xSde2aaSbaaWqaaiabdMgaPbqabaaaaaaakiabc6caUiaaxMaacaWLjaWaaeWaaeaacqaIXaqmcqaI2aGnaiaawIcacaGLPaaaaaa@74BD@

In the notation of eqn. (14) and by taking the logarithm, the Haldane relationship can be expressed as

ln⁡kleq=ln⁡k+lcat−ln⁡k−lcat+∑inilln⁡kliM.     (17)
 MathType@MTEF@5@5@+=feaafiart1ev1aaatCvAUfKttLearuWrP9MDH5MBPbIqV92AaeXatLxBI9gBaebbnrfifHhDYfgasaacH8akY=wiFfYdH8Gipec8Eeeu0xXdbba9frFj0=OqFfea0dXdd9vqai=hGuQ8kuc9pgc9s8qqaq=dirpe0xb9q8qiLsFr0=vr0=vr0dc8meaabaqaciaacaGaaeqabaqabeGadaaakeaacyGGSbaBcqGGUbGBcqWGRbWAdaqhaaWcbaGaemiBaWgabaacbaGae8xzauMae8xCaehaaOGaeyypa0JagiiBaWMaeiOBa4Maem4AaS2aa0baaSqaaiabgUcaRiabdYgaSbqaaiab=ngaJjab=fgaHjab=rha0baakiabgkHiTiGbcYgaSjabc6gaUjabdUgaRnaaDaaaleaacqGHsislcqWGSbaBaeaacqWFJbWycqWFHbqycqWF0baDaaGccqGHRaWkdaaeqbqaaiabd6gaUnaaBaaaleaacqWGPbqAcqWGSbaBaeqaaaqaaiabdMgaPbqab0GaeyyeIuoakiGbcYgaSjabc6gaUjabdUgaRnaaDaaaleaacqWGSbaBcqWGPbqAaeaacqWFnbqtaaGccqGGUaGlcaWLjaGaaCzcamaabmaabaGaeGymaeJaeG4naCdacaGLOaGaayzkaaaaaa@62E2@

For each reaction, this relationship constitutes a constraint for the kinetic parameters within the reaction. In addition, each equilibrium constant obeys

ln⁡kleq=−∑inil Gi(0)/(RT),     (18)
 MathType@MTEF@5@5@+=feaafiart1ev1aaatCvAUfKttLearuWrP9MDH5MBPbIqV92AaeXatLxBI9gBaebbnrfifHhDYfgasaacH8akY=wiFfYdH8Gipec8Eeeu0xXdbba9frFj0=OqFfea0dXdd9vqai=hGuQ8kuc9pgc9s8qqaq=dirpe0xb9q8qiLsFr0=vr0=vr0dc8meaabaqaciaacaGaaeqabaqabeGadaaakeaacyGGSbaBcqGGUbGBcqWGRbWAdaqhaaWcbaGaemiBaWgabaacbaGae8xzauMae8xCaehaaOGaeyypa0JaeyOeI0YaaabuaeaacqWGUbGBdaWgaaWcbaGaemyAaKMaemiBaWgabeaakiabbccaGaWcbaGaemyAaKgabeqdcqGHris5aOGaem4raC0aa0baaSqaaiabdMgaPbqaaiabcIcaOiabicdaWiabcMcaPaaakiabc+caViabcIcaOiabdkfasjabdsfaujabcMcaPiabcYcaSiaaxMaacaWLjaWaaeWaaeaacqaIXaqmcqaI4aaoaiaawIcacaGLPaaaaaa@4F9B@

where Gi(0)
 MathType@MTEF@5@5@+=feaafiart1ev1aaatCvAUfKttLearuWrP9MDH5MBPbIqV92AaeXatLxBI9gBaebbnrfifHhDYfgasaacH8akY=wiFfYdH8Gipec8Eeeu0xXdbba9frFj0=OqFfea0dXdd9vqai=hGuQ8kuc9pgc9s8qqaq=dirpe0xb9q8qiLsFr0=vr0=vr0dc8meaabaqaciaacaGaaeqabaqabeGadaaakeaacqWGhbWrdaqhaaWcbaGaemyAaKgabaGaeiikaGIaeGimaaJaeiykaKcaaaaa@31EB@ is the Gibbs free energy of formation of metabolite *i *(see methods). Equations (17) and (18) imply that parameters in the entire network are coupled; an arbitrary choice can easily violate the second law of thermodynamics, which is a severe obstacle to parameter optimisation and fitting.

### Thermodynamically independent system parameters

To circumvent this problem, we introduce new, thermodynamically independent system parameters [18]. For each substance *i*, we define the dimensionless energy constant

kiG=eGi(0)/(RT)     (19)
 MathType@MTEF@5@5@+=feaafiart1ev1aaatCvAUfKttLearuWrP9MDH5MBPbIqV92AaeXatLxBI9gBaebbnrfifHhDYfgasaacH8akY=wiFfYdH8Gipec8Eeeu0xXdbba9frFj0=OqFfea0dXdd9vqai=hGuQ8kuc9pgc9s8qqaq=dirpe0xb9q8qiLsFr0=vr0=vr0dc8meaabaqaciaacaGaaeqabaqabeGadaaakeaacqWGRbWAdaqhaaWcbaGaemyAaKgabaacbaGae83raCeaaOGaeyypa0Jae8xzau2aaWbaaSqabeaacqWGhbWrdaqhaaadbaGaemyAaKgabaGaeiikaGIaeGimaaJaeiykaKcaaSGaei4la8IaeiikaGIaemOuaiLaemivaqLaeiykaKcaaOGaaCzcaiaaxMaadaqadaqaaiabigdaXiabiMda5aGaayjkaiaawMcaaaaa@4243@

with Boltzmann's gas constant *R *≈ 8.314 J/(mol K) and given absolute temperature *T*. For each reaction *l*, we define the velocity constant

klV=(k+lcat k−lcat)1/2     (20)
 MathType@MTEF@5@5@+=feaafiart1ev1aaatCvAUfKttLearuWrP9MDH5MBPbIqV92AaeXatLxBI9gBaebbnrfifHhDYfgasaacH8akY=wiFfYdH8Gipec8Eeeu0xXdbba9frFj0=OqFfea0dXdd9vqai=hGuQ8kuc9pgc9s8qqaq=dirpe0xb9q8qiLsFr0=vr0=vr0dc8meaabaqaciaacaGaaeqabaqabeGadaaakeaacqWGRbWAdaqhaaWcbaGaemiBaWgabaacbaGae8NvayfaaOGaeyypa0JaeiikaGIaem4AaS2aa0baaSqaaiabgUcaRiabdYgaSbqaaiab=ngaJjab=fgaHjab=rha0baakiabbccaGiabdUgaRnaaDaaaleaacqGHsislcqWGSbaBaeaacqWFJbWycqWFHbqycqWF0baDaaGccqGGPaqkdaahaaWcbeqaaiabigdaXiabc+caViabikdaYaaakiaaxMaacaWLjaWaaeWaaeaacqaIYaGmcqaIWaamaiaawIcacaGLPaaaaaa@4BC3@

as the geometric mean of the forward and backward turn-over rate, measured in s^-1^. From now on, we shall use the energy constants and velocity constants as model parameters and treat the equilibrium constants *k*^eq ^and the turnover rates *k*^cat ^as dependent quantities: the equilibrium constants are computed from eqn. (18), and *k*^cat ^values are chosen such that equation (17) is satisfied. Using equations (17) and (18), we can write the turnover rates k±cat
 MathType@MTEF@5@5@+=feaafiart1ev1aaatCvAUfKttLearuWrP9MDH5MBPbIqV92AaeXatLxBI9gBaebbnrfifHhDYfgasaacH8akY=wiFfYdH8Gipec8Eeeu0xXdbba9frFj0=OqFfea0dXdd9vqai=hGuQ8kuc9pgc9s8qqaq=dirpe0xb9q8qiLsFr0=vr0=vr0dc8meaabaqaciaacaGaaeqabaqabeGadaaakeaacqWGRbWAdaqhaaWcbaGaeyySaelabaacbaGae83yamMae8xyaeMae8hDaqhaaaaa@342E@ as [See Additional file 1]

k±lcat=klV∏i(kiG kliM)∓nil/2     (21)=klV(∏i(kiGkliM)nil−∏i(kiGkliM)nil+)±1/2     (22)
 MathType@MTEF@5@5@+=feaafiart1ev1aaatCvAUfKttLearuWrP9MDH5MBPbIqV92AaeXatLxBI9gBaebbnrfifHhDYfgasaacH8akY=wiFfYdH8Gipec8Eeeu0xXdbba9frFj0=OqFfea0dXdd9vqai=hGuQ8kuc9pgc9s8qqaq=dirpe0xb9q8qiLsFr0=vr0=vr0dc8meaabaqaciaacaGaaeqabaqabeGadaaakeaafaqaaeGaeaaaaeaacqWGRbWAdaqhaaWcbaGaeyySaeRaemiBaWgabaGaee4yamMaeeyyaeMaeeiDaqhaaaGcbaGaeyypa0dabaGaem4AaS2aa0baaSqaaiabdYgaSbqaaiabbAfawbaakmaarafabaGaeiikaGIaem4AaS2aa0baaSqaaiabdMgaPbqaaiabbEeahbaakiabbccaGiabdUgaRnaaDaaaleaacqWGSbaBcqWGPbqAaeaacqqGnbqtaaGccqGGPaqkdaahaaWcbeqaaiabloHiTjabd6gaUnaaBaaameaacqWGPbqAcqWGSbaBaeqaaSGaei4la8IaeGOmaidaaaqaaiabdMgaPbqab0Gaey4dIunaaOqaaiaaxMaacaWLjaWaaeWaaeaacqaIYaGmcqaIXaqmaiaawIcacaGLPaaaaeaaaeaacqGH9aqpaeaacqWGRbWAdaqhaaWcbaGaemiBaWgabaGaeeOvayfaaOWaaeWaaeaadaWcaaqaamaarafabaGaeiikaGIaem4AaS2aa0baaSqaaiabdMgaPbqaaiabbEeahbaakiabdUgaRnaaDaaaleaacqWGSbaBcqWGPbqAaeaacqqGnbqtaaGccqGGPaqkdaahaaWcbeqaaiabd6gaUnaaDaaameaacqWGPbqAcqWGSbaBaeaacqGHsislaaaaaaWcbaGaemyAaKgabeqdcqGHpis1aaGcbaWaaebuaeaacqGGOaakcqWGRbWAdaqhaaWcbaGaemyAaKgabaGaee4raCeaaOGaem4AaS2aa0baaSqaaiabdYgaSjabdMgaPbqaaiabb2eanbaakiabcMcaPmaaCaaaleqabaGaemOBa42aa0baaWqaaiabdMgaPjabdYgaSbqaaiabgUcaRaaaaaaaleaacqWGPbqAaeqaniabg+GivdaaaaGccaGLOaGaayzkaaWaaWbaaSqabeaacqGHXcqScqaIXaqmcqGGVaWlcqaIYaGmaaaakeaacaWLjaGaaCzcamaabmaabaGaeGOmaiJaeGOmaidacaGLOaGaayzkaaaaaaaa@8EEB@

**Figure 1:**
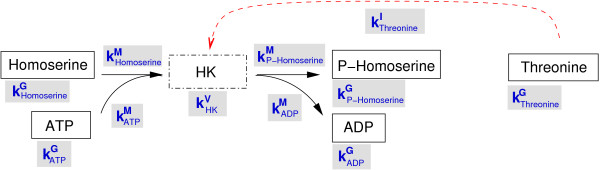
**System parameters for convenience kinetics**. The homoserine kinase reaction (HK, dotted box) transforms homoserine and ATP into O-phospho-homoserine and ADP (solid arrows). Threonine inhibits the enzyme (dotted arrow). Each node and each arrow carries one of the system parameters: each metabolite is characterised by an energy constant *k*^G^, the reaction by a velocity constant *k*^V^, and each arrow by a *k*^M ^or *k*^I ^value. The system parameters are thermodynamically independent and can assume arbitrary positive values. The turnover rates k±cat
 MathType@MTEF@5@5@+=feaafiart1ev1aaatCvAUfKttLearuWrP9MDH5MBPbIqV92AaeXatLxBI9gBaebbnrfifHhDYfgasaacH8akY=wiFfYdH8Gipec8Eeeu0xXdbba9frFj0=OqFfea0dXdd9vqai=hGuQ8kuc9pgc9s8qqaq=dirpe0xb9q8qiLsFr0=vr0=vr0dc8meaabaqaciaacaGaaeqabaqabeGadaaakeaacqWGRbWAdaqhaaWcbaGaeyySaelabaacbaGae83yamMae8xyaeMae8hDaqhaaaaa@342E@ for forward and backward direction can be computed from the system parameters.

Altogether, the convenience kinetics of a metabolic network is characterised by the system parameters listed in table 1. If a reaction network is displayed as a bipartite graph of metabolites and reactions, each of the nodes and each of the arrows in the graph is characterised by one of the parameters, as shown in Figure 1. In addition, each node can carry an enzyme concentration *E*_*l *_or a metabolite concentration *c*_*i*_; as these concentrations can fluctuate in time, we shall call them state parameters rather than system parameters. 

By taking the logarithm in both sides of eqn. (22), we obtain a linear equation between logarithmic parameters; this handy property also holds for other dependent parameters, as shown in table 2. We can express various kinetic parameters in terms of the system parameters: let *θ *denote the vector of logarithmic system parameters and **x **a vector containing various derived parameters in logarithmic form. It can be computed from *θ *by the linear relation

**Table 1:** Model parameters for convenience kinetics

Parameter	Symbol	unit	item in graph	energy interpretation
Energy constant	kiG MathType@MTEF@5@5@+=feaafiart1ev1aaatCvAUfKttLearuWrP9MDH5MBPbIqV92AaeXatLxBI9gBaebbnrfifHhDYfgasaacH8akY=wiFfYdH8Gipec8Eeeu0xXdbba9frFj0=OqFfea0dXdd9vqai=hGuQ8kuc9pgc9s8qqaq=dirpe0xb9q8qiLsFr0=vr0=vr0dc8meaabaqaciaacaGaaeqabaqabeGadaaakeaacqWGRbWAdaqhaaWcbaGaemyAaKgabaacbaGae83raCeaaaaa@30AF@	1	metabolite	metabolite formation
Velocity constant	klV MathType@MTEF@5@5@+=feaafiart1ev1aaatCvAUfKttLearuWrP9MDH5MBPbIqV92AaeXatLxBI9gBaebbnrfifHhDYfgasaacH8akY=wiFfYdH8Gipec8Eeeu0xXdbba9frFj0=OqFfea0dXdd9vqai=hGuQ8kuc9pgc9s8qqaq=dirpe0xb9q8qiLsFr0=vr0=vr0dc8meaabaqaciaacaGaaeqabaqabeGadaaakeaacqWGRbWAdaqhaaWcbaGaemiBaWgabaacbaGae8Nvayfaaaaa@30D3@	1/s	reaction	transition state
Michaelis-Menten constant	kliM MathType@MTEF@5@5@+=feaafiart1ev1aaatCvAUfKttLearuWrP9MDH5MBPbIqV92AaeXatLxBI9gBaebbnrfifHhDYfgasaacH8akY=wiFfYdH8Gipec8Eeeu0xXdbba9frFj0=OqFfea0dXdd9vqai=hGuQ8kuc9pgc9s8qqaq=dirpe0xb9q8qiLsFr0=vr0=vr0dc8meaabaqaciaacaGaaeqabaqabeGadaaakeaacqWGRbWAdaqhaaWcbaGaemiBaWMaemyAaKgabaacbaGae8xta0eaaaaa@321C@	mM	arrow reaction – substrate	substrate binding
Activation constant	kliA MathType@MTEF@5@5@+=feaafiart1ev1aaatCvAUfKttLearuWrP9MDH5MBPbIqV92AaeXatLxBI9gBaebbnrfifHhDYfgasaacH8akY=wiFfYdH8Gipec8Eeeu0xXdbba9frFj0=OqFfea0dXdd9vqai=hGuQ8kuc9pgc9s8qqaq=dirpe0xb9q8qiLsFr0=vr0=vr0dc8meaabaqaciaacaGaaeqabaqabeGadaaakeaacqWGRbWAdaqhaaWcbaGaemiBaWMaemyAaKgabaacbaGae8xqaeeaaaaa@3204@	mM	arrow reaction – activator	activator binding
Inhibition constant	kliI MathType@MTEF@5@5@+=feaafiart1ev1aaatCvAUfKttLearuWrP9MDH5MBPbIqV92AaeXatLxBI9gBaebbnrfifHhDYfgasaacH8akY=wiFfYdH8Gipec8Eeeu0xXdbba9frFj0=OqFfea0dXdd9vqai=hGuQ8kuc9pgc9s8qqaq=dirpe0xb9q8qiLsFr0=vr0=vr0dc8meaabaqaciaacaGaaeqabaqabeGadaaakeaacqWGRbWAdaqhaaWcbaGaemiBaWMaemyAaKgabaacbaGae8xsaKeaaaaa@3214@	mM	arrow reaction – inhibitor	inhibitor binding
Metabolite concentration	*c*_*i*_	mM	metabolite	
Enzyme concentration	*E*_*l*_	mM	reaction	

The system parameters (top) are thermodynamically independent. Their numerical values can be written as exp(*G/RT*) where *G *denotes either a Gibbs free energy or a difference of Gibbs free energies. The corresponding molecular processes are listed in the last column. In contrast to the system parameters, enzyme and metabolite concentrations (bottom) can easily fluctuate over time; we call them state parameters.

**Table 2:** Dependent kinetic parameters

Quantity	Symbol	unit	Formula
Gibbs free energy of formation	Gi(0) MathType@MTEF@5@5@+=feaafiart1ev1aaatCvAUfKttLearuWrP9MDH5MBPbIqV92AaeXatLxBI9gBaebbnrfifHhDYfgasaacH8akY=wiFfYdH8Gipec8Eeeu0xXdbba9frFj0=OqFfea0dXdd9vqai=hGuQ8kuc9pgc9s8qqaq=dirpe0xb9q8qiLsFr0=vr0=vr0dc8meaabaqaciaacaGaaeqabaqabeGadaaakeaacqWGhbWrdaqhaaWcbaGaemyAaKgabaGaeiikaGIaeGimaaJaeiykaKcaaaaa@31EB@	kJ/mol	Gi(0)=RTln⁡kiG MathType@MTEF@5@5@+=feaafiart1ev1aaatCvAUfKttLearuWrP9MDH5MBPbIqV92AaeXatLxBI9gBaebbnrfifHhDYfgasaacH8akY=wiFfYdH8Gipec8Eeeu0xXdbba9frFj0=OqFfea0dXdd9vqai=hGuQ8kuc9pgc9s8qqaq=dirpe0xb9q8qiLsFr0=vr0=vr0dc8meaabaqaciaacaGaaeqabaqabeGadaaakeaacqWGhbWrdaqhaaWcbaGaemyAaKgabaGaeiikaGIaeGimaaJaeiykaKcaaOGaeyypa0JaemOuaiLaemivaqLagiiBaWMaeiOBa4Maem4AaS2aa0baaSqaaiabdMgaPbqaaGqaaiab=Deahbaaaaa@3C22@
Gibbs fr. en. for substrate binding	ΔGli(0) MathType@MTEF@5@5@+=feaafiart1ev1aaatCvAUfKttLearuWrP9MDH5MBPbIqV92AaeXatLxBI9gBaebbnrfifHhDYfgasaacH8akY=wiFfYdH8Gipec8Eeeu0xXdbba9frFj0=OqFfea0dXdd9vqai=hGuQ8kuc9pgc9s8qqaq=dirpe0xb9q8qiLsFr0=vr0=vr0dc8meaabaqaciaacaGaaeqabaqabeGadaaakeaacqqHuoarcqWGhbWrdaqhaaWcbaGaemiBaWMaemyAaKgabaGaeiikaGIaeGimaaJaeiykaKcaaaaa@34B2@	kJ/mol	ΔGli(0)=RTln⁡kliM MathType@MTEF@5@5@+=feaafiart1ev1aaatCvAUfKttLearuWrP9MDH5MBPbIqV92AaeXatLxBI9gBaebbnrfifHhDYfgasaacH8akY=wiFfYdH8Gipec8Eeeu0xXdbba9frFj0=OqFfea0dXdd9vqai=hGuQ8kuc9pgc9s8qqaq=dirpe0xb9q8qiLsFr0=vr0=vr0dc8meaabaqaciaacaGaaeqabaqabeGadaaakeaacqqHuoarcqWGhbWrdaqhaaWcbaGaemiBaWMaemyAaKgabaGaeiikaGIaeGimaaJaeiykaKcaaOGaeyypa0JaemOuaiLaemivaqLagiiBaWMaeiOBa4Maem4AaS2aa0baaSqaaiabdYgaSjabdMgaPbqaaGqaaiab=1eanbaaaaa@4056@
Equilibrium constant	kleq MathType@MTEF@5@5@+=feaafiart1ev1aaatCvAUfKttLearuWrP9MDH5MBPbIqV92AaeXatLxBI9gBaebbnrfifHhDYfgasaacH8akY=wiFfYdH8Gipec8Eeeu0xXdbba9frFj0=OqFfea0dXdd9vqai=hGuQ8kuc9pgc9s8qqaq=dirpe0xb9q8qiLsFr0=vr0=vr0dc8meaabaqaciaacaGaaeqabaqabeGadaaakeaacqWGRbWAdaqhaaWcbaGaemiBaWgabaacbaGae8xzauMae8xCaehaaaaa@3258@	-	ln⁡kleq=−∑inilln⁡kiG MathType@MTEF@5@5@+=feaafiart1ev1aaatCvAUfKttLearuWrP9MDH5MBPbIqV92AaeXatLxBI9gBaebbnrfifHhDYfgasaacH8akY=wiFfYdH8Gipec8Eeeu0xXdbba9frFj0=OqFfea0dXdd9vqai=hGuQ8kuc9pgc9s8qqaq=dirpe0xb9q8qiLsFr0=vr0=vr0dc8meaabaqaciaacaGaaeqabaqabeGadaaakeaacyGGSbaBcqGGUbGBcqWGRbWAdaqhaaWcbaGaemiBaWgabaacbaGae8xzauMae8xCaehaaOGaeyypa0JaeyOeI0YaaabuaeaacqWGUbGBdaWgaaWcbaGaemyAaKMaemiBaWgabeaaaeaacqWGPbqAaeqaniabggHiLdGccyGGSbaBcqGGUbGBcqWGRbWAdaqhaaWcbaGaemyAaKgabaGae83raCeaaaaa@45A5@
Turnover rate	k±lcat MathType@MTEF@5@5@+=feaafiart1ev1aaatCvAUfKttLearuWrP9MDH5MBPbIqV92AaeXatLxBI9gBaebbnrfifHhDYfgasaacH8akY=wiFfYdH8Gipec8Eeeu0xXdbba9frFj0=OqFfea0dXdd9vqai=hGuQ8kuc9pgc9s8qqaq=dirpe0xb9q8qiLsFr0=vr0=vr0dc8meaabaqaciaacaGaaeqabaqabeGadaaakeaacqWGRbWAdaqhaaWcbaGaeyySaeRaemiBaWgabaacbaGae83yamMae8xyaeMae8hDaqhaaaaa@358F@	1/s	ln⁡k±lcat=ln⁡klV∓12∑inil(ln⁡kiG+ln⁡kliM) MathType@MTEF@5@5@+=feaafiart1ev1aaatCvAUfKttLearuWrP9MDH5MBPbIqV92AaeXatLxBI9gBaebbnrfifHhDYfgasaacH8akY=wiFfYdH8Gipec8Eeeu0xXdbba9frFj0=OqFfea0dXdd9vqai=hGuQ8kuc9pgc9s8qqaq=dirpe0xb9q8qiLsFr0=vr0=vr0dc8meaabaqaciaacaGaaeqabaqabeGadaaakeaacyGGSbaBcqGGUbGBcqWGRbWAdaqhaaWcbaGaeyySaeRaemiBaWgabaacbaGae83yamMae8xyaeMae8hDaqhaaOGaeyypa0JagiiBaWMaeiOBa4Maem4AaS2aa0baaSqaaiabdYgaSbqaaiab=zfawbaakiabloHiTnaalaaabaGaeGymaedabaGaeGOmaidaamaaqafabaGaemOBa42aaSbaaSqaaiabdMgaPjabdYgaSbqabaaabaGaemyAaKgabeqdcqGHris5aOGaeiikaGIagiiBaWMaeiOBa4Maem4AaS2aa0baaSqaaiabdMgaPbqaaiab=DeahbaakiabgUcaRiGbcYgaSjabc6gaUjabdUgaRnaaDaaaleaacqWGSbaBcqWGPbqAaeaacqWFnbqtaaGccqGGPaqkaaa@5CD7@
Maximal velocity	v±lmax⁡ MathType@MTEF@5@5@+=feaafiart1ev1aaatCvAUfKttLearuWrP9MDH5MBPbIqV92AaeXatLxBI9gBaebbnrfifHhDYfgasaacH8akY=wiFfYdH8Gipec8Eeeu0xXdbba9frFj0=OqFfea0dXdd9vqai=hGuQ8kuc9pgc9s8qqaq=dirpe0xb9q8qiLsFr0=vr0=vr0dc8meaabaqaciaacaGaaeqabaqabeGadaaakeaacqWG2bGDdaqhaaWcbaGaeyySaeRaemiBaWgabaGagiyBa0MaeiyyaeMaeiiEaGhaaaaa@35C3@	mM/s	ln⁡v±lmax=ln⁡El+ln⁡klV∓12∑inil(ln⁡kiG+ln⁡kliM) MathType@MTEF@5@5@+=feaafiart1ev1aaatCvAUfKttLearuWrP9MDH5MBPbIqV92AaeXatLxBI9gBaebbnrfifHhDYfgasaacH8akY=wiFfYdH8Gipec8Eeeu0xXdbba9frFj0=OqFfea0dXdd9vqai=hGuQ8kuc9pgc9s8qqaq=dirpe0xb9q8qiLsFr0=vr0=vr0dc8meaabaqaciaacaGaaeqabaqabeGadaaakeaacyGGSbaBcqGGUbGBcqWG2bGDdaqhaaWcbaGaeyySaeRaemiBaWgabaacbaGae8xBa0Mae8xyaeMae8hEaGhaaOGaeyypa0JagiiBaWMaeiOBa4Maemyrau0aaSbaaSqaaiabdYgaSbqabaGccqGHRaWkcyGGSbaBcqGGUbGBcqWGRbWAdaqhaaWcbaGaemiBaWgabaGae8NvayfaaOGaeS4eI02aaSaaaeaacqaIXaqmaeaacqaIYaGmaaWaaabuaeaacqWGUbGBdaWgaaWcbaGaemyAaKMaemiBaWgabeaaaeaacqWGPbqAaeqaniabggHiLdGccqGGOaakcyGGSbaBcqGGUbGBcqWGRbWAdaqhaaWcbaGaemyAaKgabaGae83raCeaaOGaey4kaSIagiiBaWMaeiOBa4Maem4AaS2aa0baaSqaaiabdYgaSjabdMgaPbqaaiab=1eanbaakiabcMcaPaaa@635B@

The logarithms of dependent parameters (and also the Gibbs free energies of formation) can be written as linear functions of the logarithmic system parameters. Equilibrium constants can have different physical units depending on the reaction stoichiometry.

x(θ)=Rθx θ.     (23)
 MathType@MTEF@5@5@+=feaafiart1ev1aaatCvAUfKttLearuWrP9MDH5MBPbIqV92AaeXatLxBI9gBaebbnrfifHhDYfgasaacH8akY=wiFfYdH8Gipec8Eeeu0xXdbba9frFj0=OqFfea0dXdd9vqai=hGuQ8kuc9pgc9s8qqaq=dirpe0xb9q8qiLsFr0=vr0=vr0dc8meaabaqaciaacaGaaeqabaqabeGadaaakeaaieqacqWF4baEcqGGOaakiiGacqGF4oqCcqGGPaqkcqGH9aqpcqWGsbGudaqhaaWcbaGae4hUdehabaGaemiEaGhaaOGaeeiiaaIae4hUdeNaeiOla4IaaCzcaiaaxMaadaqadaqaaiabikdaYiabiodaZaGaayjkaiaawMcaaaaa@3F3A@

The sensitivity matrix Rθx
 MathType@MTEF@5@5@+=feaafiart1ev1aaatCvAUfKttLearuWrP9MDH5MBPbIqV92AaeXatLxBI9gBaebbnrfifHhDYfgasaacH8akY=wiFfYdH8Gipec8Eeeu0xXdbba9frFj0=OqFfea0dXdd9vqai=hGuQ8kuc9pgc9s8qqaq=dirpe0xb9q8qiLsFr0=vr0=vr0dc8meaabaqaciaacaGaaeqabaqabeGadaaakeaacqWGsbGudaqhaaWcbaacciGae8hUdehabaGaemiEaGhaaaaa@313C@ is sparse and can be constructed easily from the network structure and the relations listed in table 2 [See Additional file 1].

By inserting the expression (22) for k±lcat
 MathType@MTEF@5@5@+=feaafiart1ev1aaatCvAUfKttLearuWrP9MDH5MBPbIqV92AaeXatLxBI9gBaebbnrfifHhDYfgasaacH8akY=wiFfYdH8Gipec8Eeeu0xXdbba9frFj0=OqFfea0dXdd9vqai=hGuQ8kuc9pgc9s8qqaq=dirpe0xb9q8qiLsFr0=vr0=vr0dc8meaabaqaciaacaGaaeqabaqabeGadaaakeaacqWGRbWAdaqhaaWcbaGaeyySaeRaemiBaWgabaacbaGae83yamMae8xyaeMae8hDaqhaaaaa@358F@ into (14), we obtain a rate law in which all parameters can be varied independently, remaining in accordance with thermodynamics. In its thermodynamically independent form, the convenience kinetics reads

vl=El∏mhA(cm,klmA)wlm+hI(cm,klmI)wlm−×klV∏ic˜linil−(k˜liM)−nil/2−∏ic˜linil+(k˜liM)nil/2∏i∑m=0nil−(c˜li)m+∏i∑m=0nil+(c˜li)m−1     (24)
 MathType@MTEF@5@5@+=feaafiart1ev1aaatCvAUfKttLearuWrP9MDH5MBPbIqV92AaeXatLxBI9gBaebbnrfifHhDYfgasaacH8akY=wiFfYdH8Gipec8Eeeu0xXdbba9frFj0=OqFfea0dXdd9vqai=hGuQ8kuc9pgc9s8qqaq=dirpe0xb9q8qiLsFr0=vr0=vr0dc8meaabaqaciaacaGaaeqabaqabeGadaaakeaafaqadeGabaaabaGaemODay3aaSbaaSqaaiabdYgaSbqabaGccqGH9aqpcqWGfbqrdaWgaaWcbaGaemiBaWgabeaakmaarafabaGaemiAaG2aaSbaaSqaaiabbgeabbqabaGccqGGOaakcqWGJbWydaWgaaWcbaGaemyBa0gabeaakiabcYcaSiabdUgaRnaaDaaaleaacqWGSbaBcqWGTbqBaeaacqqGbbqqaaGccqGGPaqkdaahaaWcbeqaaiabdEha3naaDaaameaacqWGSbaBcqWGTbqBaeaacqGHRaWkaaaaaOGaemiAaG2aaSbaaSqaaiabbMeajbqabaGccqGGOaakcqWGJbWydaWgaaWcbaGaemyBa0gabeaakiabcYcaSiabdUgaRnaaDaaaleaacqWGSbaBcqWGTbqBaeaacqqGjbqsaaGccqGGPaqkdaahaaWcbeqaaiabdEha3naaDaaameaacqWGSbaBcqWGTbqBaeaacqGHsislaaaaaaWcbaGaemyBa0gabeqdcqGHpis1aaGcbaGaey41aqRaem4AaS2aa0baaSqaaiabdYgaSbqaaiabbAfawbaakmaalaaabaWaaebuaeaacuWGJbWygaacamaaDaaaleaacqWGSbaBcqWGPbqAaeaacqWGUbGBdaqhaaadbaGaemyAaKMaemiBaWgabaGaeyOeI0caaaaaaSqaaiabdMgaPbqab0Gaey4dIunakiabcIcaOiqbdUgaRzaaiaWaa0baaSqaaiabdYgaSjabdMgaPbqaaiabd2eanbaakiabcMcaPmaaCaaaleqabaGaeyOeI0IaemOBa42aaSbaaWqaaiabdMgaPjabdYgaSbqabaWccqGGVaWlcqaIYaGmaaGccqGHsisldaqeqbqaaiqbdogaJzaaiaWaa0baaSqaaiabdYgaSjabdMgaPbqaaiabd6gaUnaaDaaameaacqWGPbqAcqWGSbaBaeaacqGHRaWkaaaaaaWcbaGaemyAaKgabeqdcqGHpis1aOGaeiikaGIafm4AaSMbaGaadaqhaaWcbaGaemiBaWMaemyAaKgabaGaemyta0eaaOGaeiykaKYaaWbaaSqabeaacqWGUbGBdaWgaaadbaGaemyAaKMaemiBaWgabeaaliabc+caViabikdaYaaaaOqaamaarafabaWaaabCaeaacqGGOaakcuWGJbWygaacamaaBaaaleaacqWGSbaBcqWGPbqAaeqaaOGaeiykaKYaaWbaaSqabeaacqWGTbqBaaaabaGaemyBa0Maeyypa0JaeGimaadabaGaemOBa42aa0baaWqaaiabdMgaPjabdYgaSbqaaiabgkHiTaaaa0GaeyyeIuoakiabgUcaRmaarafabaWaaabCaeaacqGGOaakcuWGJbWygaacamaaBaaaleaacqWGSbaBcqWGPbqAaeqaaOGaeiykaKYaaWbaaSqabeaacqWGTbqBaaaabaGaemyBa0Maeyypa0JaeGimaadabaGaemOBa42aa0baaWqaaiabdMgaPjabdYgaSbqaaiabgUcaRaaaa0GaeyyeIuoakiabgkHiTiabigdaXaWcbaGaemyAaKgabeqdcqGHpis1aaWcbaGaemyAaKgabeqdcqGHpis1aaaaaaGccaWLjaGaaCzcamaabmaabaGaeGOmaiJaeGinaqdacaGLOaGaayzkaaaaaa@CD6E@

with the abbreviations c˜li=ci/kliM
 MathType@MTEF@5@5@+=feaafiart1ev1aaatCvAUfKttLearuWrP9MDH5MBPbIqV92AaeXatLxBI9gBaebbnrfifHhDYfgasaacH8akY=wiFfYdH8Gipec8Eeeu0xXdbba9frFj0=OqFfea0dXdd9vqai=hGuQ8kuc9pgc9s8qqaq=dirpe0xb9q8qiLsFr0=vr0=vr0dc8meaabaqaciaacaGaaeqabaqabeGadaaakeaacuWGJbWygaacamaaBaaaleaacqWGSbaBcqWGPbqAaeqaaOGaeyypa0Jaem4yam2aaSbaaSqaaiabdMgaPbqabaGccqGGVaWlcqWGRbWAdaqhaaWcbaGaemiBaWMaemyAaKgabaacbaGae8xta0eaaaaa@3B38@ and k˜liM=kiGkliM
 MathType@MTEF@5@5@+=feaafiart1ev1aaatCvAUfKttLearuWrP9MDH5MBPbIqV92AaeXatLxBI9gBaebbnrfifHhDYfgasaacH8akY=wiFfYdH8Gipec8Eeeu0xXdbba9frFj0=OqFfea0dXdd9vqai=hGuQ8kuc9pgc9s8qqaq=dirpe0xb9q8qiLsFr0=vr0=vr0dc8meaabaqaciaacaGaaeqabaqabeGadaaakeaacuWGRbWAgaacamaaDaaaleaacqWGSbaBcqWGPbqAaeaaieaacqWFnbqtaaGccqGH9aqpcqWGRbWAdaqhaaWcbaGaemyAaKgabaGae83raCeaaOGaem4AaS2aa0baaSqaaiabdYgaSjabdMgaPbqaaiab=1eanbaaaaa@3CA6@. Special cases for some simple stoichiometries are listed in table 3.

**Table 3:** Convenience rate laws for common reaction stoichiometries

Reaction formula	Rate law	Turnover rates k±cat MathType@MTEF@5@5@+=feaafiart1ev1aaatCvAUfKttLearuWrP9MDH5MBPbIqV92AaeXatLxBI9gBaebbnrfifHhDYfgasaacH8akY=wiFfYdH8Gipec8Eeeu0xXdbba9frFj0=OqFfea0dXdd9vqai=hGuQ8kuc9pgc9s8qqaq=dirpe0xb9q8qiLsFr0=vr0=vr0dc8meaabaqaciaacaGaaeqabaqabeGadaaakeaacqWGRbWAdaqhaaWcbaGaeyySaelabaacbaGae83yamMae8xyaeMae8hDaqhaaaaa@342E@	Irreversible
A ↔ B	k+cata˜−k−catb˜1+a˜+b˜ MathType@MTEF@5@5@+=feaafiart1ev1aaatCvAUfKttLearuWrP9MDH5MBPbIqV92AaeXatLxBI9gBaebbnrfifHhDYfgasaacH8akY=wiFfYdH8Gipec8Eeeu0xXdbba9frFj0=OqFfea0dXdd9vqai=hGuQ8kuc9pgc9s8qqaq=dirpe0xb9q8qiLsFr0=vr0=vr0dc8meaabaqaciaacaGaaeqabaqabeGadaaakeaadaWcaaqaaiabdUgaRnaaDaaaleaacqGHRaWkaeaaieaacqWFJbWycqWFHbqycqWF0baDaaGccuWGHbqygaacaiabgkHiTiabdUgaRnaaDaaaleaacqGHsislaeaacqWFJbWycqWFHbqycqWF0baDaaGccuWGIbGygaacaaqaaiabigdaXiabgUcaRiqbdggaHzaaiaGaey4kaSIafmOyaiMbaGaaaaaaaa@42CB@	kV(k˜AMk˜BM)±1/2 MathType@MTEF@5@5@+=feaafiart1ev1aaatCvAUfKttLearuWrP9MDH5MBPbIqV92AaeXatLxBI9gBaebbnrfifHhDYfgasaacH8akY=wiFfYdH8Gipec8Eeeu0xXdbba9frFj0=OqFfea0dXdd9vqai=hGuQ8kuc9pgc9s8qqaq=dirpe0xb9q8qiLsFr0=vr0=vr0dc8meaabaqaciaacaGaaeqabaqabeGadaaakeaacqWGRbWAdaahaaWcbeqaaGqaaiab=zfawbaakiabcIcaOmaalaaabaGafm4AaSMbaGaadaqhaaWcbaGae8xqaeeabaGae8xta0eaaaGcbaGafm4AaSMbaGaadaqhaaWcbaGae8NqaieabaGae8xta0eaaaaakiabcMcaPmaaCaaaleqabaGaeyySaeRaeGymaeJaei4la8IaeGOmaidaaaaa@3DB9@	k+cata˜1+a˜ MathType@MTEF@5@5@+=feaafiart1ev1aaatCvAUfKttLearuWrP9MDH5MBPbIqV92AaeXatLxBI9gBaebbnrfifHhDYfgasaacH8akY=wiFfYdH8Gipec8Eeeu0xXdbba9frFj0=OqFfea0dXdd9vqai=hGuQ8kuc9pgc9s8qqaq=dirpe0xb9q8qiLsFr0=vr0=vr0dc8meaabaqaciaacaGaaeqabaqabeGadaaakeaadaWcaaqaaiabdUgaRnaaDaaaleaacqGHRaWkaeaaieaacqWFJbWycqWFHbqycqWF0baDaaGccuWGHbqygaacaaqaaiabigdaXiabgUcaRiqbdggaHzaaiaaaaaaa@37C2@
A + X ↔ B	k+cata˜x˜−k−catb˜1+a˜+x˜+a˜x˜+b˜ MathType@MTEF@5@5@+=feaafiart1ev1aaatCvAUfKttLearuWrP9MDH5MBPbIqV92AaeXatLxBI9gBaebbnrfifHhDYfgasaacH8akY=wiFfYdH8Gipec8Eeeu0xXdbba9frFj0=OqFfea0dXdd9vqai=hGuQ8kuc9pgc9s8qqaq=dirpe0xb9q8qiLsFr0=vr0=vr0dc8meaabaqaciaacaGaaeqabaqabeGadaaakeaadaWcaaqaaiabdUgaRnaaDaaaleaacqGHRaWkaeaaieaacqWFJbWycqWFHbqycqWF0baDaaGccuWGHbqygaacaiqbdIha4zaaiaGaeyOeI0Iaem4AaS2aa0baaSqaaiabgkHiTaqaaiab=ngaJjab=fgaHjab=rha0baakiqbdkgaIzaaiaaabaGaeGymaeJaey4kaSIafmyyaeMbaGaacqGHRaWkcuWG4baEgaacaiabgUcaRiqbdggaHzaaiaGafmiEaGNbaGaacqGHRaWkcuWGIbGygaacaaaaaaa@4A81@	kV(k˜AMk˜XMk˜BM)±1/2 MathType@MTEF@5@5@+=feaafiart1ev1aaatCvAUfKttLearuWrP9MDH5MBPbIqV92AaeXatLxBI9gBaebbnrfifHhDYfgasaacH8akY=wiFfYdH8Gipec8Eeeu0xXdbba9frFj0=OqFfea0dXdd9vqai=hGuQ8kuc9pgc9s8qqaq=dirpe0xb9q8qiLsFr0=vr0=vr0dc8meaabaqaciaacaGaaeqabaqabeGadaaakeaacqWGRbWAdaahaaWcbeqaaGqaaiab=zfawbaakiabcIcaOmaalaaabaGafm4AaSMbaGaadaqhaaWcbaGae8xqaeeabaGae8xta0eaaOGafm4AaSMbaGaadaqhaaWcbaGae8hwaGfabaGae8xta0eaaaGcbaGafm4AaSMbaGaadaqhaaWcbaGae8NqaieabaGae8xta0eaaaaakiabcMcaPmaaCaaaleqabaGaeyySaeRaeGymaeJaei4la8IaeGOmaidaaaaa@41B2@	k+cata˜x˜1+a˜+x˜+a˜x˜ MathType@MTEF@5@5@+=feaafiart1ev1aaatCvAUfKttLearuWrP9MDH5MBPbIqV92AaeXatLxBI9gBaebbnrfifHhDYfgasaacH8akY=wiFfYdH8Gipec8Eeeu0xXdbba9frFj0=OqFfea0dXdd9vqai=hGuQ8kuc9pgc9s8qqaq=dirpe0xb9q8qiLsFr0=vr0=vr0dc8meaabaqaciaacaGaaeqabaqabeGadaaakeaadaWcaaqaaiabdUgaRnaaDaaaleaacqGHRaWkaeaaieaacqWFJbWycqWFHbqycqWF0baDaaGccuWGHbqygaacaiqbdIha4zaaiaaabaGaeGymaeJaey4kaSIafmyyaeMbaGaacqGHRaWkcuWG4baEgaacaiabgUcaRiqbdggaHzaaiaGafmiEaGNbaGaaaaaaaa@3F78@
A + X ↔ B + Y	k+cata˜x˜−k−catb˜y˜1+a˜+x˜+a˜x˜+b˜+y˜+b˜y˜ MathType@MTEF@5@5@+=feaafiart1ev1aaatCvAUfKttLearuWrP9MDH5MBPbIqV92AaeXatLxBI9gBaebbnrfifHhDYfgasaacH8akY=wiFfYdH8Gipec8Eeeu0xXdbba9frFj0=OqFfea0dXdd9vqai=hGuQ8kuc9pgc9s8qqaq=dirpe0xb9q8qiLsFr0=vr0=vr0dc8meaabaqaciaacaGaaeqabaqabeGadaaakeaadaWcaaqaaiabdUgaRnaaDaaaleaacqGHRaWkaeaaieaacqWFJbWycqWFHbqycqWF0baDaaGccuWGHbqygaacaiqbdIha4zaaiaGaeyOeI0Iaem4AaS2aa0baaSqaaiabgkHiTaqaaiab=ngaJjab=fgaHjab=rha0baakiqbdkgaIzaaiaGafmyEaKNbaGaaaeaacqaIXaqmcqGHRaWkcuWGHbqygaacaiabgUcaRiqbdIha4zaaiaGaey4kaSIafmyyaeMbaGaacuWG4baEgaacaiabgUcaRiqbdkgaIzaaiaGaey4kaSIafmyEaKNbaGaacqGHRaWkcuWGIbGygaacaiqbdMha5zaaiaaaaaaa@523F@	kV(k˜AMk˜XMk˜BMk˜YM)±1/2 MathType@MTEF@5@5@+=feaafiart1ev1aaatCvAUfKttLearuWrP9MDH5MBPbIqV92AaeXatLxBI9gBaebbnrfifHhDYfgasaacH8akY=wiFfYdH8Gipec8Eeeu0xXdbba9frFj0=OqFfea0dXdd9vqai=hGuQ8kuc9pgc9s8qqaq=dirpe0xb9q8qiLsFr0=vr0=vr0dc8meaabaqaciaacaGaaeqabaqabeGadaaakeaacqWGRbWAdaahaaWcbeqaaGqaaiab=zfawbaakiabcIcaOmaalaaabaGafm4AaSMbaGaadaqhaaWcbaGae8xqaeeabaGae8xta0eaaOGafm4AaSMbaGaadaqhaaWcbaGae8hwaGfabaGae8xta0eaaaGcbaGafm4AaSMbaGaadaqhaaWcbaGae8NqaieabaGae8xta0eaaOGafm4AaSMbaGaadaqhaaWcbaGae8xwaKfabaGae8xta0eaaaaakiabcMcaPmaaCaaaleqabaGaeyySaeRaeGymaeJaei4la8IaeGOmaidaaaaa@45AD@	k+cata˜x˜1+a˜+x˜+a˜x˜ MathType@MTEF@5@5@+=feaafiart1ev1aaatCvAUfKttLearuWrP9MDH5MBPbIqV92AaeXatLxBI9gBaebbnrfifHhDYfgasaacH8akY=wiFfYdH8Gipec8Eeeu0xXdbba9frFj0=OqFfea0dXdd9vqai=hGuQ8kuc9pgc9s8qqaq=dirpe0xb9q8qiLsFr0=vr0=vr0dc8meaabaqaciaacaGaaeqabaqabeGadaaakeaadaWcaaqaaiabdUgaRnaaDaaaleaacqGHRaWkaeaaieaacqWFJbWycqWFHbqycqWF0baDaaGccuWGHbqygaacaiqbdIha4zaaiaaabaGaeGymaeJaey4kaSIafmyyaeMbaGaacqGHRaWkcuWG4baEgaacaiabgUcaRiqbdggaHzaaiaGafmiEaGNbaGaaaaaaaa@3F78@
2 A ↔ B	k+cata˜2−k−catb˜1+a˜+a˜2+b˜ MathType@MTEF@5@5@+=feaafiart1ev1aaatCvAUfKttLearuWrP9MDH5MBPbIqV92AaeXatLxBI9gBaebbnrfifHhDYfgasaacH8akY=wiFfYdH8Gipec8Eeeu0xXdbba9frFj0=OqFfea0dXdd9vqai=hGuQ8kuc9pgc9s8qqaq=dirpe0xb9q8qiLsFr0=vr0=vr0dc8meaabaqaciaacaGaaeqabaqabeGadaaakeaadaWcaaqaaiabdUgaRnaaDaaaleaacqGHRaWkaeaaieaacqWFJbWycqWFHbqycqWF0baDaaGccuWGHbqygaacamaaCaaaleqabaGaeGOmaidaaOGaeyOeI0Iaem4AaS2aa0baaSqaaiabgkHiTaqaaiab=ngaJjab=fgaHjab=rha0baakiqbdkgaIzaaiaaabaGaeGymaeJaey4kaSIafmyyaeMbaGaacqGHRaWkcuWGHbqygaacamaaCaaaleqabaGaeGOmaidaaOGaey4kaSIafmOyaiMbaGaaaaaaaa@4759@	kV((k˜AM)2k˜BM)±1/2 MathType@MTEF@5@5@+=feaafiart1ev1aaatCvAUfKttLearuWrP9MDH5MBPbIqV92AaeXatLxBI9gBaebbnrfifHhDYfgasaacH8akY=wiFfYdH8Gipec8Eeeu0xXdbba9frFj0=OqFfea0dXdd9vqai=hGuQ8kuc9pgc9s8qqaq=dirpe0xb9q8qiLsFr0=vr0=vr0dc8meaabaqaciaacaGaaeqabaqabeGadaaakeaacqWGRbWAdaahaaWcbeqaaGqaaiab=zfawbaakiabcIcaOmaalaaabaGaeiikaGIafm4AaSMbaGaadaqhaaWcbaGae8xqaeeabaGae8xta0eaaOGaeiykaKYaaWbaaSqabeaacqaIYaGmaaaakeaacuWGRbWAgaacamaaDaaaleaacqWFcbGqaeaacqWFnbqtaaaaaOGaeiykaKYaaWbaaSqabeaacqGHXcqScqaIXaqmcqGGVaWlcqaIYaGmaaaaaa@4094@	k+cata˜21+a˜+a˜2 MathType@MTEF@5@5@+=feaafiart1ev1aaatCvAUfKttLearuWrP9MDH5MBPbIqV92AaeXatLxBI9gBaebbnrfifHhDYfgasaacH8akY=wiFfYdH8Gipec8Eeeu0xXdbba9frFj0=OqFfea0dXdd9vqai=hGuQ8kuc9pgc9s8qqaq=dirpe0xb9q8qiLsFr0=vr0=vr0dc8meaabaqaciaacaGaaeqabaqabeGadaaakeaadaWcaaqaaiabdUgaRnaaDaaaleaacqGHRaWkaeaacqqGJbWycqqGHbqycqqG0baDaaGccuWGHbqygaacamaaCaaaleqabaGaeGOmaidaaaGcbaGaeGymaeJaey4kaSIafmyyaeMbaGaacqGHRaWkcuWGHbqygaacamaaCaaaleqabaGaeGOmaidaaaaaaaa@3C43@
2 A ↔ B + Y	k+cata˜2−k−catb˜y˜1+a˜+a˜2+b˜+y˜+b˜y˜ MathType@MTEF@5@5@+=feaafiart1ev1aaatCvAUfKttLearuWrP9MDH5MBPbIqV92AaeXatLxBI9gBaebbnrfifHhDYfgasaacH8akY=wiFfYdH8Gipec8Eeeu0xXdbba9frFj0=OqFfea0dXdd9vqai=hGuQ8kuc9pgc9s8qqaq=dirpe0xb9q8qiLsFr0=vr0=vr0dc8meaabaqaciaacaGaaeqabaqabeGadaaakeaadaWcaaqaaiabdUgaRnaaDaaaleaacqGHRaWkaeaaieaacqWFJbWycqWFHbqycqWF0baDaaGccuWGHbqygaacamaaCaaaleqabaGaeGOmaidaaOGaeyOeI0Iaem4AaS2aa0baaSqaaiabgkHiTaqaaiab=ngaJjab=fgaHjab=rha0baakiqbdkgaIzaaiaGafmyEaKNbaGaaaeaacqaIXaqmcqGHRaWkcuWGHbqygaacaiabgUcaRiqbdggaHzaaiaWaaWbaaSqabeaacqaIYaGmaaGccqGHRaWkcuWGIbGygaacaiabgUcaRiqbdMha5zaaiaGaey4kaSIafmOyaiMbaGaacuWG5bqEgaacaaaaaaa@4F17@	kV((k˜AM)2k˜BMk˜YM)±1/2 MathType@MTEF@5@5@+=feaafiart1ev1aaatCvAUfKttLearuWrP9MDH5MBPbIqV92AaeXatLxBI9gBaebbnrfifHhDYfgasaacH8akY=wiFfYdH8Gipec8Eeeu0xXdbba9frFj0=OqFfea0dXdd9vqai=hGuQ8kuc9pgc9s8qqaq=dirpe0xb9q8qiLsFr0=vr0=vr0dc8meaabaqaciaacaGaaeqabaqabeGadaaakeaacqWGRbWAdaahaaWcbeqaaGqaaiab=zfawbaakiabcIcaOmaalaaabaGaeiikaGIafm4AaSMbaGaadaqhaaWcbaGae8xqaeeabaGae8xta0eaaOGaeiykaKYaaWbaaSqabeaacqaIYaGmaaaakeaacuWGRbWAgaacamaaDaaaleaacqWFcbGqaeaacqWFnbqtaaGccuWGRbWAgaacamaaDaaaleaacqWFzbqwaeaacqWFnbqtaaaaaOGaeiykaKYaaWbaaSqabeaacqGHXcqScqaIXaqmcqGGVaWlcqaIYaGmaaaaaa@448F@	k+cata˜21+a˜+a˜2 MathType@MTEF@5@5@+=feaafiart1ev1aaatCvAUfKttLearuWrP9MDH5MBPbIqV92AaeXatLxBI9gBaebbnrfifHhDYfgasaacH8akY=wiFfYdH8Gipec8Eeeu0xXdbba9frFj0=OqFfea0dXdd9vqai=hGuQ8kuc9pgc9s8qqaq=dirpe0xb9q8qiLsFr0=vr0=vr0dc8meaabaqaciaacaGaaeqabaqabeGadaaakeaadaWcaaqaaiabdUgaRnaaDaaaleaacqGHRaWkaeaacqqGJbWycqqGHbqycqqG0baDaaGccuWGHbqygaacamaaCaaaleqabaGaeGOmaidaaaGcbaGaeGymaeJaey4kaSIafmyyaeMbaGaacqGHRaWkcuWGHbqygaacamaaCaaaleqabaGaeGOmaidaaaaaaaa@3C43@
2 A + X ↔ B	k+cata˜2x˜−k−catb˜(1+a˜+a˜2)(1+x˜)+b˜ MathType@MTEF@5@5@+=feaafiart1ev1aaatCvAUfKttLearuWrP9MDH5MBPbIqV92AaeXatLxBI9gBaebbnrfifHhDYfgasaacH8akY=wiFfYdH8Gipec8Eeeu0xXdbba9frFj0=OqFfea0dXdd9vqai=hGuQ8kuc9pgc9s8qqaq=dirpe0xb9q8qiLsFr0=vr0=vr0dc8meaabaqaciaacaGaaeqabaqabeGadaaakeaadaWcaaqaaiabdUgaRnaaDaaaleaacqGHRaWkaeaaieaacqWFJbWycqWFHbqycqWF0baDaaGccuWGHbqygaacamaaCaaaleqabaGaeGOmaidaaOGafmiEaGNbaGaacqGHsislcqWGRbWAdaqhaaWcbaGaeyOeI0cabaGae83yamMae8xyaeMae8hDaqhaaOGafmOyaiMbaGaaaeaacqGGOaakcqaIXaqmcqGHRaWkcuWGHbqygaacaiabgUcaRiqbdggaHzaaiaWaaWbaaSqabeaacqaIYaGmaaGccqGGPaqkcqGGOaakcqaIXaqmcqGHRaWkcuWG4baEgaacaiabcMcaPiabgUcaRiqbdkgaIzaaiaaaaaaa@4F9F@	kV((k˜AM)2k˜XMk˜BM)±1/2 MathType@MTEF@5@5@+=feaafiart1ev1aaatCvAUfKttLearuWrP9MDH5MBPbIqV92AaeXatLxBI9gBaebbnrfifHhDYfgasaacH8akY=wiFfYdH8Gipec8Eeeu0xXdbba9frFj0=OqFfea0dXdd9vqai=hGuQ8kuc9pgc9s8qqaq=dirpe0xb9q8qiLsFr0=vr0=vr0dc8meaabaqaciaacaGaaeqabaqabeGadaaakeaacqWGRbWAdaahaaWcbeqaaGqaaiab=zfawbaakiabcIcaOmaalaaabaGaeiikaGIafm4AaSMbaGaadaqhaaWcbaGae8xqaeeabaGae8xta0eaaOGaeiykaKYaaWbaaSqabeaacqaIYaGmaaGccuWGRbWAgaacamaaDaaaleaacqWFybawaeaacqWFnbqtaaaakeaacuWGRbWAgaacamaaDaaaleaacqWFcbGqaeaacqWFnbqtaaaaaOGaeiykaKYaaWbaaSqabeaacqGHXcqScqaIXaqmcqGGVaWlcqaIYaGmaaaaaa@448D@	k+cata˜2x˜(1+a˜+a˜2)(1+x˜) MathType@MTEF@5@5@+=feaafiart1ev1aaatCvAUfKttLearuWrP9MDH5MBPbIqV92AaeXatLxBI9gBaebbnrfifHhDYfgasaacH8akY=wiFfYdH8Gipec8Eeeu0xXdbba9frFj0=OqFfea0dXdd9vqai=hGuQ8kuc9pgc9s8qqaq=dirpe0xb9q8qiLsFr0=vr0=vr0dc8meaabaqaciaacaGaaeqabaqabeGadaaakeaadaWcaaqaaiabdUgaRnaaDaaaleaacqGHRaWkaeaacqqGJbWycqqGHbqycqqG0baDaaGccuWGHbqygaacamaaCaaaleqabaGaeGOmaidaaOGafmiEaGNbaGaaaeaacqGGOaakcqaIXaqmcqGHRaWkcuWGHbqygaacaiabgUcaRiqbdggaHzaaiaWaaWbaaSqabeaacqaIYaGmaaGccqGGPaqkcqGGOaakcqaIXaqmcqGHRaWkcuWG4baEgaacaiabcMcaPaaaaaa@4493@

The rate laws follow from the enzyme mechanism and reflect the reaction stoichiometry; for each case, the thermodynamically independent expression of the turnover rates and the irreversible form are also shown. We use the shortcuts a˜=a/kAM
 MathType@MTEF@5@5@+=feaafiart1ev1aaatCvAUfKttLearuWrP9MDH5MBPbIqV92AaeXatLxBI9gBaebbnrfifHhDYfgasaacH8akY=wiFfYdH8Gipec8Eeeu0xXdbba9frFj0=OqFfea0dXdd9vqai=hGuQ8kuc9pgc9s8qqaq=dirpe0xb9q8qiLsFr0=vr0=vr0dc8meaabaqaciaacaGaaeqabaqabeGadaaakeaacuWGHbqygaacaiabg2da9iabdggaHjabc+caViabdUgaRnaaDaaaleaacqqGbbqqaeaacqqGnbqtaaaaaa@34F3@ and k˜AM=kAGkAM
 MathType@MTEF@5@5@+=feaafiart1ev1aaatCvAUfKttLearuWrP9MDH5MBPbIqV92AaeXatLxBI9gBaebbnrfifHhDYfgasaacH8akY=wiFfYdH8Gipec8Eeeu0xXdbba9frFj0=OqFfea0dXdd9vqai=hGuQ8kuc9pgc9s8qqaq=dirpe0xb9q8qiLsFr0=vr0=vr0dc8meaabaqaciaacaGaaeqabaqabeGadaaakeaacuWGRbWAgaacamaaDaaaleaaieaacqWFbbqqaeaacqWFnbqtaaGccqGH9aqpcqWGRbWAdaqhaaWcbaGae8xqaeeabaGae83raCeaaOGaem4AaS2aa0baaSqaaiab=feabbqaaiab=1eanbaaaaa@38E8@ for metabolite A and analogous shortcuts for the other metabolites. For brevity, the prefactors for enzyme concentration and enzyme regulation are not shown.

### Energy interpretation of the parameters

All system parameters can be expressed in terms of Gibbs free energies: the *k*^M^, *k*^A^, and *k*^I ^values represent binding energies, and the energy constants *k*^G ^are defined by the Gibbs free energy of formation. Finally, we can also write the velocity constants as

kV=e−ΔGtr(0)/(RT)s−1.     (25)
 MathType@MTEF@5@5@+=feaafiart1ev1aaatCvAUfKttLearuWrP9MDH5MBPbIqV92AaeXatLxBI9gBaebbnrfifHhDYfgasaacH8akY=wiFfYdH8Gipec8Eeeu0xXdbba9frFj0=OqFfea0dXdd9vqai=hGuQ8kuc9pgc9s8qqaq=dirpe0xb9q8qiLsFr0=vr0=vr0dc8meaabaqaciaacaGaaeqabaqabeGadaaakeaacqWGRbWAdaahaaWcbeqaaGqaaiab=zfawbaakiabg2da9iab=vgaLnaaCaaaleqabaGaeyOeI0IaeuiLdqKaem4raC0aa0baaWqaaiab=rha0jab=jhaYbqaaiabcIcaOiabicdaWiabcMcaPaaaliabc+caViabcIcaOiabdkfasjabdsfaujabcMcaPaaakiab=nhaZnaaCaaaleqabaGaeyOeI0IaeGymaedaaOGaeiOla4IaaCzcaiaaxMaadaqadaqaaiabikdaYiabiwda1aGaayjkaiaawMcaaaaa@4931@

To illustrate the meaning of the energy ΔGtr(0)
 MathType@MTEF@5@5@+=feaafiart1ev1aaatCvAUfKttLearuWrP9MDH5MBPbIqV92AaeXatLxBI9gBaebbnrfifHhDYfgasaacH8akY=wiFfYdH8Gipec8Eeeu0xXdbba9frFj0=OqFfea0dXdd9vqai=hGuQ8kuc9pgc9s8qqaq=dirpe0xb9q8qiLsFr0=vr0=vr0dc8meaabaqaciaacaGaaeqabaqabeGadaaakeaacqqHuoarcqWGhbWrdaqhaaWcbaacbaGae8hDaqNae8NCaihabaGaeiikaGIaeGimaaJaeiykaKcaaaaa@34D5@, we consider again the bimolecular enzymatic mechanism: in transition state theory [15], the rate constants between the substrate and product complex are formally written as

k+cat=e−(Gtr(0)−GEAX(0))/(RT)s−1k−cat=e−(Gtr(0)−GEBY(0))/(RT)s−1,     (26)
 MathType@MTEF@5@5@+=feaafiart1ev1aaatCvAUfKttLearuWrP9MDH5MBPbIqV92AaeXatLxBI9gBaebbnrfifHhDYfgasaacH8akY=wiFfYdH8Gipec8Eeeu0xXdbba9frFj0=OqFfea0dXdd9vqai=hGuQ8kuc9pgc9s8qqaq=dirpe0xb9q8qiLsFr0=vr0=vr0dc8meaabaqaciaacaGaaeqabaqabeGadaaakeaafaqaaeGadaaabaGaem4AaS2aa0baaSqaaiabgUcaRaqaaGqaaiab=ngaJjab=fgaHjab=rha0baaaOqaaiabg2da9aqaaiabdwgaLnaaCaaaleqabaGaeyOeI0IaeiikaGIaem4raC0aa0baaWqaaiab=rha0jab=jhaYbqaamaabmaabaGaeGimaadacaGLOaGaayzkaaaaaSGaeyOeI0Iaem4raC0aa0baaWqaaiabdweafnaaBaaabaGae8xqaeKae8hwaGfabeaaaeaadaqadaqaaiabicdaWaGaayjkaiaawMcaaaaaliabcMcaPiabc+caVmaabmaabaGaemOuaiLaemivaqfacaGLOaGaayzkaaaaaOGaem4Cam3aaWbaaSqabeaacqGHsislcqaIXaqmaaaakeaacqWGRbWAdaqhaaWcbaGaeyOeI0cabaGae83yamMae8xyaeMae8hDaqhaaaGcbaGaeyypa0dabaGaemyzau2aaWbaaSqabeaacqGHsislcqGGOaakcqWGhbWrdaqhaaadbaGae8hDaqNae8NCaihabaWaaeWaaeaacqaIWaamaiaawIcacaGLPaaaaaWccqGHsislcqWGhbWrdaqhaaadbaGaemyrau0aaSbaaeaacqWFcbGqcqWFzbqwaeqaaaqaamaabmaabaGaeGimaadacaGLOaGaayzkaaaaaSGaeiykaKIaei4la8YaaeWaaeaacqWGsbGucqWGubavaiaawIcacaGLPaaaaaGccqWGZbWCdaahaaWcbeqaaiabgkHiTiabigdaXaaakiabcYcaSaaacaWLjaGaaCzcamaabmaabaGaeGOmaiJaeGOnaydacaGLOaGaayzkaaaaaa@77DD@

where the quantities *G*^(0) ^denote Gibbs free energies of formation for the substrate complex *E*_AX_, the product complex *E*_BY_, and a hypothetical transition state *E*^tr ^that has to be crossed on the way from *E*_AX _to *E*_BY_. By inserting eqn. (26) into the definition (20) and defining an energy barrier ΔGtr(0)=Gtr(0)−12(GEAX(0)+GEBY(0))
 MathType@MTEF@5@5@+=feaafiart1ev1aaatCvAUfKttLearuWrP9MDH5MBPbIqV92AaeXatLxBI9gBaebbnrfifHhDYfgasaacH8akY=wiFfYdH8Gipec8Eeeu0xXdbba9frFj0=OqFfea0dXdd9vqai=hGuQ8kuc9pgc9s8qqaq=dirpe0xb9q8qiLsFr0=vr0=vr0dc8meaabaqaciaacaGaaeqabaqabeGadaaakeaacqqHuoarcqWGhbWrdaqhaaWcbaacbaGae8hDaqNae8NCaihabaWaaeWaaeaacqaIWaamaiaawIcacaGLPaaaaaGccqGH9aqpcqWGhbWrdaqhaaWcbaGae8hDaqNae8NCaihabaWaaeWaaeaacqaIWaamaiaawIcacaGLPaaaaaGccqGHsisldaWcaaqaaiabigdaXaqaaiabikdaYaaadaqadaqaaiabdEeahnaaDaaaleaacqWGfbqrdaWgaaadbaGae8xqaeKae8hwaGfabeaaaSqaamaabmaabaGaeGimaadacaGLOaGaayzkaaaaaOGaey4kaSIaem4raC0aa0baaSqaaiabdweafnaaBaaameaacqWFcbGqcqWFzbqwaeqaaaWcbaWaaeWaaeaacqaIWaamaiaawIcacaGLPaaaaaaakiaawIcacaGLPaaaaaa@503D@, we obtain eqn. (25).

### Independent equilibrium constants as system parameters

We introduced the energy constants kiG
 MathType@MTEF@5@5@+=feaafiart1ev1aaatCvAUfKttLearuWrP9MDH5MBPbIqV92AaeXatLxBI9gBaebbnrfifHhDYfgasaacH8akY=wiFfYdH8Gipec8Eeeu0xXdbba9frFj0=OqFfea0dXdd9vqai=hGuQ8kuc9pgc9s8qqaq=dirpe0xb9q8qiLsFr0=vr0=vr0dc8meaabaqaciaacaGaaeqabaqabeGadaaakeaacqWGRbWAdaqhaaWcbaGaemyAaKgabaacbaGae83raCeaaaaa@30AF@ as model parameters for two reasons: first, they provide a consistent way to describe the equilibrium constants; secondly, if Gibbs free energies of formation are known from experiments, they can be used for fitting the energy constants and will thus contribute to a good choice of equilibrium constants. However, if no such data are available, the second reason becomes redundant, and a different choice of the system parameters may be appropriate: instead of the energy constants, we employ a set of independent equilibrium constants. If the stoichiometric matrix *N *has full column rank, then the equilibrium constants are independent anyway because for given **k**^eq^, eqn. (18) can always be satisfied by some choice of the Gi(0)
 MathType@MTEF@5@5@+=feaafiart1ev1aaatCvAUfKttLearuWrP9MDH5MBPbIqV92AaeXatLxBI9gBaebbnrfifHhDYfgasaacH8akY=wiFfYdH8Gipec8Eeeu0xXdbba9frFj0=OqFfea0dXdd9vqai=hGuQ8kuc9pgc9s8qqaq=dirpe0xb9q8qiLsFr0=vr0=vr0dc8meaabaqaciaacaGaaeqabaqabeGadaaakeaacqWGhbWrdaqhaaWcbaGaemyAaKgabaGaeiikaGIaeGimaaJaeiykaKcaaaaa@31EB@; in this case, the equilibrium constants can be directly used as model parameters. Otherwise, we can choose a set of reactions with the following property: their equilibrium constants (collected in a vector **k**^ind^) are thermodynamically independent, and they determine all other equilibrium constants in the model via a linear equation

ln keq=Rindeq ln kind.     (27)
 MathType@MTEF@5@5@+=feaafiart1ev1aaatCvAUfKttLearuWrP9MDH5MBPbIqV92AaeXatLxBI9gBaebbnrfifHhDYfgasaacH8akY=wiFfYdH8Gipec8Eeeu0xXdbba9frFj0=OqFfea0dXdd9vqai=hGuQ8kuc9pgc9s8qqaq=dirpe0xb9q8qiLsFr0=vr0=vr0dc8meaabaqaciaacaGaaeqabaqabeGadaaakeaaieaacqWFSbaBcqWFUbGBcqqGGaaiieqacqGFRbWAdaahaaWcbeqaaiabbwgaLjabbghaXbaakiabg2da9iabdkfasnaaDaaaleaacqWFPbqAcqWFUbGBcqWFKbazaeaacqWFLbqzcqWFXbqCaaGccqqGGaaicqqGSbaBcqqGUbGBcqqGGaaicqGFRbWAdaahaaWcbeqaaiabbMgaPjabb6gaUjabbsgaKbaakiabc6caUiaaxMaacaWLjaWaaeWaaeaacqaIYaGmcqaI3aWnaiaawIcacaGLPaaaaaa@4D3F@

The choice of independent reactions and the computation of Rindeq
 MathType@MTEF@5@5@+=feaafiart1ev1aaatCvAUfKttLearuWrP9MDH5MBPbIqV92AaeXatLxBI9gBaebbnrfifHhDYfgasaacH8akY=wiFfYdH8Gipec8Eeeu0xXdbba9frFj0=OqFfea0dXdd9vqai=hGuQ8kuc9pgc9s8qqaq=dirpe0xb9q8qiLsFr0=vr0=vr0dc8meaabaqaciaacaGaaeqabaqabeGadaaakeaacqWGsbGudaqhaaWcbaacbaGae8xAaKMae8NBa4Mae8hzaqgabaGae8xzauMae8xCaehaaaaa@34CA@ are explained in the methods section. Given the equilibrium and velocity constants, the turnover rates can be expressed as

k±lcat=klV(kleq)±1/2,     (28)
 MathType@MTEF@5@5@+=feaafiart1ev1aaatCvAUfKttLearuWrP9MDH5MBPbIqV92AaeXatLxBI9gBaebbnrfifHhDYfgasaacH8akY=wiFfYdH8Gipec8Eeeu0xXdbba9frFj0=OqFfea0dXdd9vqai=hGuQ8kuc9pgc9s8qqaq=dirpe0xb9q8qiLsFr0=vr0=vr0dc8meaabaqaciaacaGaaeqabaqabeGadaaakeaacqWGRbWAdaqhaaWcbaGaeyySaeRaemiBaWgabaacbaGae83yamMae8xyaeMae8hDaqhaaOGaeyypa0Jaem4AaS2aa0baaSqaaiabdYgaSbqaaiab=zfawbaakmaabmaabaGaem4AaS2aa0baaSqaaiabdYgaSbqaaiab=vgaLjab=fhaXbaaaOGaayjkaiaawMcaamaaCaaaleqabaGaeyySaeRaeGymaeJaei4la8IaeGOmaidaaOGaeiilaWIaaCzcaiaaxMaadaqadaqaaiabikdaYiabiIda4aGaayjkaiaawMcaaaaa@4C87@

or equivalently as

ln⁡ k±lcat=ln⁡ klV±12ln⁡ kleq     (29)
 MathType@MTEF@5@5@+=feaafiart1ev1aaatCvAUfKttLearuWrP9MDH5MBPbIqV92AaeXatLxBI9gBaebbnrfifHhDYfgasaacH8akY=wiFfYdH8Gipec8Eeeu0xXdbba9frFj0=OqFfea0dXdd9vqai=hGuQ8kuc9pgc9s8qqaq=dirpe0xb9q8qiLsFr0=vr0=vr0dc8meaabaqaciaacaGaaeqabaqabeGadaaakeaacyGGSbaBcqGGUbGBcqqGGaaiieGacqWFRbWAdaqhaaWcbaGaeyySaeRae8hBaWgabaGaee4yamMaeeyyaeMaeeiDaqhaaOGaeyypa0JagiiBaWMaeiOBa4MaeeiiaaIae83AaS2aa0baaSqaaiab=XgaSbqaaiabbAfawbaakiabgglaXoaalaaabaGaeGymaedabaGaeGOmaidaaiGbcYgaSjabc6gaUjabbccaGiab=TgaRnaaDaaaleaacqWFSbaBaeaacqqGLbqzcqqGXbqCaaGccaWLjaGaaCzcamaabmaabaGaeGOmaiJaeGyoaKdacaGLOaGaayzkaaaaaa@53B0@

and be inserted into eqn. (14).

### The convenience kinetics resembles other rate laws

To check whether the convenience kinetics yields any unusual results, we compared it to two established rate laws, namely the ordered and ping-pong mechanisms for bimolecular reactions. In both mechanisms, binding and dissociation occur in a fixed order:



Besides the turnover rates and *k*^M ^values, their kinetic laws also contain product inhibition constants. For the comparison, we made the simplifying (yet biologically realistic) assumption that these inhibition constants equal the respective k^M ^values, which yields the following rate laws [8]

Ordered mechanism:v=Ek+cata˜x˜−k−catb˜y˜1+a˜+x˜+a˜x˜+b˜+y˜+b˜y˜+a˜b˜+x˜y˜+a˜x˜b˜+x˜b˜y˜     (30)
 MathType@MTEF@5@5@+=feaafiart1ev1aaatCvAUfKttLearuWrP9MDH5MBPbIqV92AaeXatLxBI9gBaebbnrfifHhDYfgasaacH8akY=wiFfYdH8Gipec8Eeeu0xXdbba9frFj0=OqFfea0dXdd9vqai=hGuQ8kuc9pgc9s8qqaq=dirpe0xb9q8qiLsFr0=vr0=vr0dc8meaabaqaciaacaGaaeqabaqabeGadaaakeaafaqabeqaeaaaaeaacqqGpbWtcqqGYbGCcqqGKbazcqqGLbqzcqqGYbGCcqqGLbqzcqqGKbazcqqGGaaicqqGTbqBcqqGLbqzcqqGJbWycqqGObaAcqqGHbqycqqGUbGBcqqGPbqAcqqGZbWCcqqGTbqBcqGG6aGoaeaacqWG2bGDaeaacqGH9aqpaeaacqWGfbqrdaWcaaqaaiabdUgaRnaaDaaaleaacqGHRaWkaeaacqqGJbWycqqGHbqycqqG0baDaaGccuWGHbqygaacaiqbdIha4zaaiaGaeyOeI0Iaem4AaS2aa0baaSqaaiabgkHiTaqaaiabbogaJjabbggaHjabbsha0baakiqbdkgaIzaaiaGafmyEaKNbaGaaaeaacqaIXaqmcqGHRaWkcuWGHbqygaacaiabgUcaRiqbdIha4zaaiaGaey4kaSIafmyyaeMbaGaacuWG4baEgaacaiabgUcaRiqbdkgaIzaaiaGaey4kaSIafmyEaKNbaGaacqGHRaWkcuWGIbGygaacaiqbdMha5zaaiaGaey4kaSIafmyyaeMbaGaacuWGIbGygaacaiabgUcaRiqbdIha4zaaiaGafmyEaKNbaGaacqGHRaWkcuWGHbqygaacaiqbdIha4zaaiaGafmOyaiMbaGaacqGHRaWkcuWG4baEgaacaiqbdkgaIzaaiaGafmyEaKNbaGaaaaaaaiaaxMaacaWLjaWaaeWaaeaacqaIZaWmcqaIWaamaiaawIcacaGLPaaaaaa@83B3@

Ping-pongmechanism:v=Ek+cata˜x˜−k−catb˜y˜a˜+x˜+a˜x˜+b˜+y˜+b˜y˜+a˜b˜+x˜y˜.     (31)
 MathType@MTEF@5@5@+=feaafiart1ev1aaatCvAUfKttLearuWrP9MDH5MBPbIqV92AaeXatLxBI9gBaebbnrfifHhDYfgasaacH8akY=wiFfYdH8Gipec8Eeeu0xXdbba9frFj0=OqFfea0dXdd9vqai=hGuQ8kuc9pgc9s8qqaq=dirpe0xb9q8qiLsFr0=vr0=vr0dc8meaabaqaciaacaGaaeqabaqabeGadaaakeaacqqGqbaucqqGPbqAcqqGUbGBcqqGNbWzcqqGTaqlcqqGWbaCcqqGVbWBcqqGUbGBcqqGNbWzfaqabeqaeaaaaeaacqqGTbqBcqqGLbqzcqqGJbWycqqGObaAcqqGHbqycqqGUbGBcqqGPbqAcqqGZbWCcqqGTbqBcqGG6aGoaeaacqWG2bGDaeaacqGH9aqpaeaacqWGfbqrdaWcaaqaaiabdUgaRnaaDaaaleaacqGHRaWkaeaacqqGJbWycqqGHbqycqqG0baDaaGccuWGHbqygaacaiqbdIha4zaaiaGaeyOeI0Iaem4AaS2aa0baaSqaaiabgkHiTaqaaiabbogaJjabbggaHjabbsha0baakiqbdkgaIzaaiaGafmyEaKNbaGaaaeaacuWGHbqygaacaiabgUcaRiqbdIha4zaaiaGaey4kaSIafmyyaeMbaGaacuWG4baEgaacaiabgUcaRiqbdkgaIzaaiaGaey4kaSIafmyEaKNbaGaacqGHRaWkcuWGIbGygaacaiqbdMha5zaaiaGaey4kaSIafmyyaeMbaGaacuWGIbGygaacaiabgUcaRiqbdIha4zaaiaGafmyEaKNbaGaaaaGaeiOla4caaiaaxMaacaWLjaWaaeWaaeaacqaIZaWmcqaIXaqmaiaawIcacaGLPaaaaaa@79F2@

**Figure 2:**
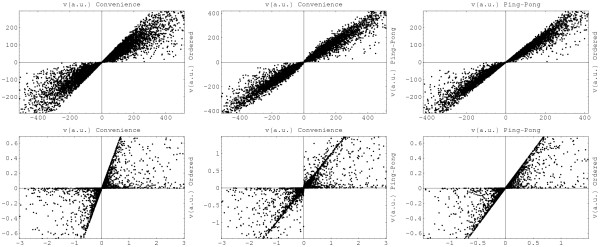
**Comparison of ordered, ping-pong, and convenience kinetics**. Kinetic parameters and reactant concentrations were drawn from random distributions; each of the rate laws yields different reaction velocities. Top: concentrations and parameters were drawn from a uniform distribution. The scatter plots show the results from convenience versus ordered kinetics (left, linear correlation coefficient *R *= 0.94), convenience versus ping-pong kinetics (centre, *R *= 0.98), and ping-pong versus ordered kinetics (right, *R *= 0.98). The similarity between convenience and ping-pong kinetics is higher than between ping-pong and the ordered kinetics. Bottom: a log-uniform distribution yields different distributions and smaller correlations, but a similar qualitative result. Again, the plots show convenience versus ordered kinetics (left, *R *= 0.73), convenience versus ping-pong kinetics (centre, *R *= 0.90), and ping-pong versus ordered kinetics (right, *R *= 0.84).

In contrast to the convenience rate law (8), the denominators contain mixed terms between substrates and products, and in the ping-pong kinetics, the term +1 is missing. The ordered mechanism yields smaller reaction rates than the ping-pong and the convenience kinetics because its denominator is always larger. To compare the three rate laws, we sampled metabolite concentrations and *k*^M ^values from a random distribution and computed the resulting reaction velocities. Parameters and concentrations were independently sampled from a uniform distribution in the interval [0.001, 1000] and from a log-uniform distribution on the same interval. Figure 2 shows scatter plots between reaction velocities computed from the different rate laws. For the uniform distribution, the results from convenience kinetics resemble those from ordered and ping-pong kinetics; they are about as similar as the ordered and ping-pong kinetics. With the log-uniform distribution, the correlations between all three kinetics become smaller, and ping-pong kinetics is more similar to convenience than to ordered kinetics. We conclude that erroneously choosing convenience kinetics instead of the other kinetic laws is just as risky as a wrong choice between the two other mechanisms.

### Parameter estimation

The parameters in convenience kinetics – the independent and the resulting dependent ones – can be measured in experiments. The linear relationship (23) makes it particularly easy to use such experimental values for parameter fitting: given a metabolic network, we mine the literature for thermodynamic and kinetic data, in particular Gibbs free energies of formation, reaction Gibbs free energies, equilibrium constants, *k*^M ^values, *k*^I ^values, *k*^A ^values, and turnover rates, and merge their logarithms in a large vector x*. The vector can contain multiple values for a parameter, it can contain thermodynamically dependent parameters, and of course, many parameters from the model will be missing. We try to determine a vector *θ *of logarithmic system parameters that yields a good match between the resulting parameter predictions **x **(*θ*) and the data x*. Solving x* ≈ Rθx
 MathType@MTEF@5@5@+=feaafiart1ev1aaatCvAUfKttLearuWrP9MDH5MBPbIqV92AaeXatLxBI9gBaebbnrfifHhDYfgasaacH8akY=wiFfYdH8Gipec8Eeeu0xXdbba9frFj0=OqFfea0dXdd9vqai=hGuQ8kuc9pgc9s8qqaq=dirpe0xb9q8qiLsFr0=vr0=vr0dc8meaabaqaciaacaGaaeqabaqabeGadaaakeaacqWGsbGudaqhaaWcbaacciGae8hUdehabaGaemiEaGhaaaaa@313C@*θ *for *θ *by the method of least squares yields an estimate of the system parameters. Using eqn. (23) again, consistent values of all kinetic parameters can be computed from the estimated system parameters. Contradictions in the original data are resolved; in addition, we can employ a prior distribution representing typical parameter ranges to compensate for missing data. A more general estimation procedure, which can also integrate measured metabolic concentrations and fluxes, is described in the companion article [19].

## Discussion

Convenience kinetics can be used for modelling biochemical systems in a simple and standardised way. In contrast to ad-hoc rate laws such as linlog or generalised mass-action kinetics, the convenience kinetics is biochemically justified as a direct generalisation of the Michaelis-Menten kinetics; it is saturable and allows for activation and inhibition of the enzyme. The parameters *k*^M^, *k*^A^, and *k*^I ^represent concentrations that lead to half-maximal (or in general, (1 + *α*_*i*_)^-1 ^-maximal) effects: the *k*^M ^values also indicate the threshold between low substrate concentrations that lead to linear kinetics and high concentrations at which the enzyme works in saturation.

The convenience kinetics represents a rapid-equilibrium random-order enzyme mechanism. When all substrates are bound, they are converted in a single step into the products, which then dissociate from the enzyme. The *k*^M^, *k*^A^, and *k*^I ^values represent dissociation constants between the enzyme and the reactant or modifier, while *k*^V ^represents the velocity of the transformation step. The system parameters also provide a sensible basis for describing variability in cell populations: the Gibbs free energies of formation depend on the composition of the cytosol, for instance its pH and temperature, and can be expected to show small, possibly correlated variations. The remaining parameters reflect interaction energies, which depend on the enzyme's amino acid sequence; we can expect that these energies vary between cells, and probably more independently than, for instance, the forward and backward turnover rates.

The convenience kinetics does not differ strikingly from established kinetic laws: in a comparison with the ordered and ping-pong mechanisms, the convenience kinetics resembled the ping-pong mechanism, and the similarity between them was greater than that between the ordered and ping-pong mechanisms. Mathematically, the three rate laws differ in their denominators: in convenience kinetics, we find all combinations of substrate concentrations and all combinations of product concentrations, but no mixed terms containing both substrate and product concentrations. The single terms reflect the reactant complexes formed by the enzyme.

The second concern of this paper was the incorporation of thermodynamic constraints: in pathway-based methods [21-23], proper treatment of the Gibbs free energies yields constraints on the flux directions; in our kinetic models, it leads to linear dependencies between the logarithmic parameters. To eliminate these constraints, we express the equilibrium constants **k**^eq ^by Gibbs free energies of formation or we choose a set of independent equilibrium constants. This trick is of course not limited to the convenience kinetics: independent parameters and equations of the form (23) can also be used with many other kinetic laws, in particular those that share the denominator of the convenience rate law; also other modes of activation and inhibition can be treated in the same manner as long as the modifiers do not affect the chemical equilibrium.

The choice of rate laws and parameter values is a main bottleneck in kinetic modelling. Standard rate laws such as the convenience kinetics can facilitate the automatic construction and fitting of large kinetic models. For transcriptional regulation, a general saturable law has been proposed [24]. For metabolic systems, the convenience kinetics may be a mathematically handy and biologically plausible choice whenever the detailed enzymatic mechanism is unknown. Estimates of model parameters can be obtained by integration of kinetic, metabolic, and proteomic data as described in the companion article [19].

## Conclusion

In kinetic modelling, every chemical reaction has to be characterised by a kinetic law and by the corresponding parameters. The convenience kinetics applies to arbitrary reaction stoichiometries and captures biologically relevant behaviour (saturation, activation, inhibition) with a small number of free parameters. It represents a simple molecular reaction mechanism in which substrates bind rapidly and in random order to the enzyme, without energetic interaction between the binding sites. The same holds for the dissociation of products.

For reactions with a single substrate and a single product, the convenience kinetics equals the well-known Michaelis-Menten kinetics. By introducing a set of thermodynamically independent system parameters, we obtained a form of the rate law that ensures thermodynamic correctness and is notably suited for parameter fitting and optimisation.

## Methods

### Basic notions for metabolic models

The structure of a metabolic network is defined by the lists of metabolites and reactions and by two structural matrices, *N *and *W*. The coefficients *n*_*il *_contained in the stoichiometric matrix *N *describe how many molecules of type *i *are produced in reaction *l*; negative elements describe consumption of molecules. The elements of the regulation matrix *W *describe enzyme regulation between metabolites *i *and enzymes *l*: *w*_*li *_= 1 indicates activation, *w*_*li *_= -1 represents inhibition, and *w*_*li *_= 0 no interaction.

In the setting of deterministic differential equations, the substance concentrations in a biochemical system follow the balance equations

ddtc=N v(c,k).     (32)
 MathType@MTEF@5@5@+=feaafiart1ev1aaatCvAUfKttLearuWrP9MDH5MBPbIqV92AaeXatLxBI9gBaebbnrfifHhDYfgasaacH8akY=wiFfYdH8Gipec8Eeeu0xXdbba9frFj0=OqFfea0dXdd9vqai=hGuQ8kuc9pgc9s8qqaq=dirpe0xb9q8qiLsFr0=vr0=vr0dc8meaabaqaciaacaGaaeqabaqabeGadaaakeaadaWcaaqaaGqaaiab=rgaKbqaaiab=rgaKjabdsha0baaieqacqGFJbWycqGH9aqpcqWGobGtcqqGGaaicqGF2bGDdaqadaqaaiab+ngaJjabbYcaSiab+TgaRbGaayjkaiaawMcaaiabc6caUiaaxMaacaWLjaWaaeWaaeaacqaIZaWmcqaIYaGmaiaawIcacaGLPaaaaaa@4129@

The vectors **c**, **v**, and **k **contain the metabolite concentrations (in mM), reaction velocities (in mM/s), and system parameters, respectively. External or buffered metabolites with fixed concentrations are contained in the parameter vector **k**.

To relate activation and inhibition (as stated in *W*) to the reaction kinetics, we first assume a hypothetical kinetic law without regulation; in this law, the reaction velocity depends only on the substrate and product concentrations. In the real rate law, a metabolite is an activator if (i) it increases the rate although it is not a reactant, or (ii) it increases the rate more strongly than it would by just being a reactant. Inhibition is defined analogously.

### Thermodynamical properties

The kinetic laws *v*_*l*_(**c**, **k**) are constrained by fundamental thermodynamic laws that relate the metabolite concentrations in steady state to molecular energies [15]. A single reaction event of reaction *l *changes the Gibbs free energy of the system by

ΔGl=∑inil μi     (33)
 MathType@MTEF@5@5@+=feaafiart1ev1aaatCvAUfKttLearuWrP9MDH5MBPbIqV92AaeXatLxBI9gBaebbnrfifHhDYfgasaacH8akY=wiFfYdH8Gipec8Eeeu0xXdbba9frFj0=OqFfea0dXdd9vqai=hGuQ8kuc9pgc9s8qqaq=dirpe0xb9q8qiLsFr0=vr0=vr0dc8meaabaqaciaacaGaaeqabaqabeGadaaakeaacqqHuoarcqWGhbWrdaWgaaWcbaGaemiBaWgabeaakiabg2da9maaqafabaGaemOBa42aaSbaaSqaaiabdMgaPjabdYgaSbqabaaabaGaemyAaKgabeqdcqGHris5aOGaeeiiaaccciGae8hVd02aaSbaaSqaaiabdMgaPbqabaGccaWLjaGaaCzcamaabmaabaGaeG4mamJaeG4mamdacaGLOaGaayzkaaaaaa@425A@

where the sum runs over all metabolites and *μ*_*i *_denotes the chemical potential of metabolite *i *(in kJ/mol). In an ideal mixed phase at pressure *P *and absolute temperature *T*, the chemical potential of substance *i *with concentration *c*_*i *_reads

μi(P,T,ci)=μi(0)(P,T)+RT ln ci     (34)
 MathType@MTEF@5@5@+=feaafiart1ev1aaatCvAUfKttLearuWrP9MDH5MBPbIqV92AaeXatLxBI9gBaebbnrfifHhDYfgasaacH8akY=wiFfYdH8Gipec8Eeeu0xXdbba9frFj0=OqFfea0dXdd9vqai=hGuQ8kuc9pgc9s8qqaq=dirpe0xb9q8qiLsFr0=vr0=vr0dc8meaabaqaciaacaGaaeqabaqabeGadaaakeaaiiGacqWF8oqBdaWgaaWcbaGaemyAaKgabeaakmaabmaabaGaemiuaaLaeiilaWIaemivaqLaeiilaWIaem4yam2aaSbaaSqaaiabdMgaPbqabaaakiaawIcacaGLPaaacqGH9aqpcqWF8oqBdaqhaaWcbaGaemyAaKgabaWaaeWaaeaacqaIWaamaiaawIcacaGLPaaaaaGcdaqadaqaaiabdcfaqjabcYcaSiabdsfaubGaayjkaiaawMcaaiabgUcaRiabdkfasjabdsfaujabbccaGiabbYgaSjabb6gaUjabbccaGGqaciab+ngaJnaaBaaaleaacqGFPbqAaeqaaOGaaCzcaiaaxMaadaqadaqaaiabiodaZiabisda0aGaayjkaiaawMcaaaaa@5328@

where μi(0)
 MathType@MTEF@5@5@+=feaafiart1ev1aaatCvAUfKttLearuWrP9MDH5MBPbIqV92AaeXatLxBI9gBaebbnrfifHhDYfgasaacH8akY=wiFfYdH8Gipec8Eeeu0xXdbba9frFj0=OqFfea0dXdd9vqai=hGuQ8kuc9pgc9s8qqaq=dirpe0xb9q8qiLsFr0=vr0=vr0dc8meaabaqaciaacaGaaeqabaqabeGadaaakeaaiiGacqWF8oqBdaqhaaWcbaGaemyAaKgabaWaaeWaaeaacqaIWaamaiaawIcacaGLPaaaaaaaaa@3268@ denotes the chemical potential of the pure substance at infinite dilution, and *R *≈ 8.314 J/(mol K) is Boltzmann's gas constant. In (34), the *c*_*i *_are dimensionless numbers denoting concentrations in mM. In real mixed phases, there would be an additional term +*RT *ln fi0
 MathType@MTEF@5@5@+=feaafiart1ev1aaatCvAUfKttLearuWrP9MDH5MBPbIqV92AaeXatLxBI9gBaebbnrfifHhDYfgasaacH8akY=wiFfYdH8Gipec8Eeeu0xXdbba9frFj0=OqFfea0dXdd9vqai=hGuQ8kuc9pgc9s8qqaq=dirpe0xb9q8qiLsFr0=vr0=vr0dc8meaabaqaciaacaGaaeqabaqabeGadaaakeaacqWGMbGzdaqhaaWcbaGaemyAaKgabaGaeGimaadaaaaa@3077@ with the activity coefficient *f*_*i*_. We neglect this term, assuming an ideal mixed phase without mixture effects on volume or energy; we also neglect effects of changing pressure or electric charges.

The equilibrium constant of reaction *l *is defined as

kleq=∏i(cieq)nil     (35)
 MathType@MTEF@5@5@+=feaafiart1ev1aaatCvAUfKttLearuWrP9MDH5MBPbIqV92AaeXatLxBI9gBaebbnrfifHhDYfgasaacH8akY=wiFfYdH8Gipec8Eeeu0xXdbba9frFj0=OqFfea0dXdd9vqai=hGuQ8kuc9pgc9s8qqaq=dirpe0xb9q8qiLsFr0=vr0=vr0dc8meaabaqaciaacaGaaeqabaqabeGadaaakeaacqWGRbWAdaqhaaWcbaGaemiBaWgabaacbaGae8xzauMae8xCaehaaOGaeyypa0ZaaebuaeaadaqadaqaaiabdogaJnaaDaaaleaacqWGPbqAaeaacqWFLbqzcqWFXbqCaaaakiaawIcacaGLPaaaaSqaaiabdMgaPbqab0Gaey4dIunakmaaCaaaleqabaGaemOBa42aaSbaaWqaaiabdMgaPjabdYgaSbqabaaaaOGaaCzcaiaaxMaadaqadaqaaiabiodaZiabiwda1aGaayjkaiaawMcaaaaa@473D@

where **c**^eq ^is the vector of metabolite concentrations in a chemical equilibrium state. According to the second law of thermodynamics, the equilibrium state of a chemical system is characterised by a minimum of the Gibbs free energy. This implies that each chemical reaction in equilibrium satisfies Δ*G*_*l *_= 0. From eqs. (33), (34), and (35) follows

ln⁡kleq=∑inilln⁡cieq=−ΔGl(0)R T,     (36)
 MathType@MTEF@5@5@+=feaafiart1ev1aaatCvAUfKttLearuWrP9MDH5MBPbIqV92AaeXatLxBI9gBaebbnrfifHhDYfgasaacH8akY=wiFfYdH8Gipec8Eeeu0xXdbba9frFj0=OqFfea0dXdd9vqai=hGuQ8kuc9pgc9s8qqaq=dirpe0xb9q8qiLsFr0=vr0=vr0dc8meaabaqaciaacaGaaeqabaqabeGadaaakeaacyGGSbaBcqGGUbGBcqWGRbWAdaqhaaWcbaGaemiBaWgabaacbaGae8xzauMae8xCaehaaOGaeyypa0ZaaabuaeaacqWGUbGBdaWgaaWcbaGaemyAaKMaemiBaWgabeaakiGbcYgaSjabc6gaUjabdogaJnaaDaaaleaacqWGPbqAaeaacqWFLbqzcqWFXbqCaaGccqGH9aqpcqGHsisldaWcaaqaaiabfs5aejabdEeahnaaDaaaleaacqWGSbaBaeaadaqadaqaaiabicdaWaGaayjkaiaawMcaaaaaaOqaaiabdkfasjabbccaGiabdsfaubaacqGGSaalcaWLjaGaaCzcamaabmaabaGaeG4mamJaeGOnaydacaGLOaGaayzkaaaaleaacqWGPbqAaeqaniabggHiLdaaaa@57AF@

where ΔGl(0)=∑inil μi(0)(P,T)
 MathType@MTEF@5@5@+=feaafiart1ev1aaatCvAUfKttLearuWrP9MDH5MBPbIqV92AaeXatLxBI9gBaebbnrfifHhDYfgasaacH8akY=wiFfYdH8Gipec8Eeeu0xXdbba9frFj0=OqFfea0dXdd9vqai=hGuQ8kuc9pgc9s8qqaq=dirpe0xb9q8qiLsFr0=vr0=vr0dc8meaabaqaciaacaGaaeqabaqabeGadaaakeaacqqHuoarcqWGhbWrdaqhaaWcbaGaemiBaWgabaWaaeWaaeaacqaIWaamaiaawIcacaGLPaaaaaGccqGH9aqpdaaeqbqaaiabd6gaUnaaBaaaleaacqWGPbqAcqWGSbaBcqqGGaaiaeqaaGGacOGae8hVd02aa0baaSqaaiabdMgaPbqaamaabmaabaGaeGimaadacaGLOaGaayzkaaaaaaqaaiabdMgaPbqab0GaeyyeIuoakmaabmaabaGaemiuaaLaeiilaWIaemivaqfacaGLOaGaayzkaaaaaa@4758@ is called the standard reaction Gibbs free energy and the concentrations are measured in mM. It can also be expressed as

ΔGl(0)=∑inil Gi(0)     (37)
 MathType@MTEF@5@5@+=feaafiart1ev1aaatCvAUfKttLearuWrP9MDH5MBPbIqV92AaeXatLxBI9gBaebbnrfifHhDYfgasaacH8akY=wiFfYdH8Gipec8Eeeu0xXdbba9frFj0=OqFfea0dXdd9vqai=hGuQ8kuc9pgc9s8qqaq=dirpe0xb9q8qiLsFr0=vr0=vr0dc8meaabaqaciaacaGaaeqabaqabeGadaaakeaacqqHuoarcqWGhbWrdaqhaaWcbaGaemiBaWgabaWaaeWaaeaacqaIWaamaiaawIcacaGLPaaaaaGccqGH9aqpdaaeqbqaaiabd6gaUnaaBaaaleaacqWGPbqAcqWGSbaBcqqGGaaiaeqaaaqaaiabdMgaPbqab0GaeyyeIuoakiabdEeahnaaDaaaleaacqWGPbqAaeaadaqadaqaaiabicdaWaGaayjkaiaawMcaaaaakiaaxMaacaWLjaWaaeWaaeaacqaIZaWmcqaI3aWnaiaawIcacaGLPaaaaaa@46AC@

in terms of the Gibbs free energies of formation Gi(0)
 MathType@MTEF@5@5@+=feaafiart1ev1aaatCvAUfKttLearuWrP9MDH5MBPbIqV92AaeXatLxBI9gBaebbnrfifHhDYfgasaacH8akY=wiFfYdH8Gipec8Eeeu0xXdbba9frFj0=OqFfea0dXdd9vqai=hGuQ8kuc9pgc9s8qqaq=dirpe0xb9q8qiLsFr0=vr0=vr0dc8meaabaqaciaacaGaaeqabaqabeGadaaakeaacqWGhbWrdaqhaaWcbaGaemyAaKgabaGaeiikaGIaeGimaaJaeiykaKcaaaaa@31EB@ for a standard state, typically *P *= 1.015 bar and *T *= 298.15 K. Equations (36) and (37) constitute the relation (18) between equilibrium constants and the Gibbs free energies of formation.

### Selection of independent equilibrium constants

Dependencies between equilibrium constants can be treated in a similar manner to the linear dependencies that constitute the conservation relations between metabolites [25]. To choose a set of reactions with independent equilibrium constants – for brevity, we shall call them independent reactions – we collect a maximal number of linearly independent columns of *N *and join them in a matrix N˜
 MathType@MTEF@5@5@+=feaafiart1ev1aaatCvAUfKttLearuWrP9MDH5MBPbIqV92AaeXatLxBI9gBaebbnrfifHhDYfgasaacH8akY=wiFfYdH8Gipec8Eeeu0xXdbba9frFj0=OqFfea0dXdd9vqai=hGuQ8kuc9pgc9s8qqaq=dirpe0xb9q8qiLsFr0=vr0=vr0dc8meaabaqaciaacaGaaeqabaqabeGadaaakeaacuWGobGtgaacaaaa@2DE0@. The chosen columns correspond to the independent reactions, and their choice need not be unique. By construction, N˜
 MathType@MTEF@5@5@+=feaafiart1ev1aaatCvAUfKttLearuWrP9MDH5MBPbIqV92AaeXatLxBI9gBaebbnrfifHhDYfgasaacH8akY=wiFfYdH8Gipec8Eeeu0xXdbba9frFj0=OqFfea0dXdd9vqai=hGuQ8kuc9pgc9s8qqaq=dirpe0xb9q8qiLsFr0=vr0=vr0dc8meaabaqaciaacaGaaeqabaqabeGadaaakeaacuWGobGtgaacaaaa@2DE0@ has full column rank, and we can split *N *into a matrix product *N *= N˜
 MathType@MTEF@5@5@+=feaafiart1ev1aaatCvAUfKttLearuWrP9MDH5MBPbIqV92AaeXatLxBI9gBaebbnrfifHhDYfgasaacH8akY=wiFfYdH8Gipec8Eeeu0xXdbba9frFj0=OqFfea0dXdd9vqai=hGuQ8kuc9pgc9s8qqaq=dirpe0xb9q8qiLsFr0=vr0=vr0dc8meaabaqaciaacaGaaeqabaqabeGadaaakeaacuWGobGtgaacaaaa@2DE0@L˜
 MathType@MTEF@5@5@+=feaafiart1ev1aaatCvAUfKttLearuWrP9MDH5MBPbIqV92AaeXatLxBI9gBaebbnrfifHhDYfgasaacH8akY=wiFfYdH8Gipec8Eeeu0xXdbba9frFj0=OqFfea0dXdd9vqai=hGuQ8kuc9pgc9s8qqaq=dirpe0xb9q8qiLsFr0=vr0=vr0dc8meaabaqaciaacaGaaeqabaqabeGadaaakeaacuWGmbatgaacaaaa@2DDC@, by analogy to the splitting *N *= *L N*_R _that is used in metabolic control analysis to remove dependent metabolites.

To be thermodynamically feasible, the equilibrium constants have to satisfy eqn. (18) or, in vector form,

ln **k**^eq ^= -*N*^T ^ln **k**^G ^    (38)

for at least one choice of the vector **k**^G^. Let us first assume that **k**^G ^is given; then the equilibrium constants of the independent reactions read

ln **k**^ind ^= -N˜
 MathType@MTEF@5@5@+=feaafiart1ev1aaatCvAUfKttLearuWrP9MDH5MBPbIqV92AaeXatLxBI9gBaebbnrfifHhDYfgasaacH8akY=wiFfYdH8Gipec8Eeeu0xXdbba9frFj0=OqFfea0dXdd9vqai=hGuQ8kuc9pgc9s8qqaq=dirpe0xb9q8qiLsFr0=vr0=vr0dc8meaabaqaciaacaGaaeqabaqabeGadaaakeaacuWGobGtgaacaaaa@2DE0@^T ^ln **k**^G^,     (39)

and with the definition

Rindeq=L˜T     (40)
 MathType@MTEF@5@5@+=feaafiart1ev1aaatCvAUfKttLearuWrP9MDH5MBPbIqV92AaeXatLxBI9gBaebbnrfifHhDYfgasaacH8akY=wiFfYdH8Gipec8Eeeu0xXdbba9frFj0=OqFfea0dXdd9vqai=hGuQ8kuc9pgc9s8qqaq=dirpe0xb9q8qiLsFr0=vr0=vr0dc8meaabaqaciaacaGaaeqabaqabeGadaaakeaacqWGsbGudaqhaaWcbaacbaGae8xAaKMae8NBa4Mae8hzaqgabaGae8xzauMae8xCaehaaOGaeyypa0JafmitaWKbaGaadaahaaWcbeqaaiab=rfaubaakiaaxMaacaWLjaWaaeWaaeaacqaI0aancqaIWaamaiaawIcacaGLPaaaaaa@3D1F@

we can write

ln⁡keq=−L˜TN˜Tln⁡kG=Rindeqln⁡kind.     (41)
 MathType@MTEF@5@5@+=feaafiart1ev1aaatCvAUfKttLearuWrP9MDH5MBPbIqV92AaeXatLxBI9gBaebbnrfifHhDYfgasaacH8akY=wiFfYdH8Gipec8Eeeu0xXdbba9frFj0=OqFfea0dXdd9vqai=hGuQ8kuc9pgc9s8qqaq=dirpe0xb9q8qiLsFr0=vr0=vr0dc8meaabaqaciaacaGaaeqabaqabeGadaaakeaacyGGSbaBcqGGUbGBieqacqWFRbWAdaahaaWcbeqaaGqaaiab+vgaLjab+fhaXbaakiabg2da9iabgkHiTiqbdYeamzaaiaWaaWbaaSqabeaacqGFubavaaGccuWGobGtgaacamaaCaaaleqabaGae4hvaqfaaOGagiiBaWMaeiOBa4Mae83AaS2aaWbaaSqabeaacqGFhbWraaGccqGH9aqpcqWGsbGudaqhaaWcbaGae4xAaKMae4NBa4Mae4hzaqgabaGae4xzauMae4xCaehaaOGagiiBaWMaeiOBa4Mae83AaS2aaWbaaSqabeaacqGFPbqAcqGFUbGBcqGFKbazaaGccqGGUaGlcaWLjaGaaCzcamaabmaabaGaeGinaqJaeGymaedacaGLOaGaayzkaaaaaa@5763@

Hence, eqn. (27) is satisfied and the matrix Rindeq
 MathType@MTEF@5@5@+=feaafiart1ev1aaatCvAUfKttLearuWrP9MDH5MBPbIqV92AaeXatLxBI9gBaebbnrfifHhDYfgasaacH8akY=wiFfYdH8Gipec8Eeeu0xXdbba9frFj0=OqFfea0dXdd9vqai=hGuQ8kuc9pgc9s8qqaq=dirpe0xb9q8qiLsFr0=vr0=vr0dc8meaabaqaciaacaGaaeqabaqabeGadaaakeaacqWGsbGudaqhaaWcbaacbaGae8xAaKMae8NBa4Mae8hzaqgabaGae8xzauMae8xCaehaaaaa@34CA@ is known. It remains to be shown that the equilibrium constants contained in **k**^ind ^are indeed thermodynamically independent; or in other words, that for any vector ln **k**^ind^, there exists a vector **k**^G ^such that eqn. (39) holds; and this is indeed the case because N˜
 MathType@MTEF@5@5@+=feaafiart1ev1aaatCvAUfKttLearuWrP9MDH5MBPbIqV92AaeXatLxBI9gBaebbnrfifHhDYfgasaacH8akY=wiFfYdH8Gipec8Eeeu0xXdbba9frFj0=OqFfea0dXdd9vqai=hGuQ8kuc9pgc9s8qqaq=dirpe0xb9q8qiLsFr0=vr0=vr0dc8meaabaqaciaacaGaaeqabaqabeGadaaakeaacuWGobGtgaacaaaa@2DE0@^T ^has full row rank.

## Competing interests

The authors declare that they have no competing interests.

## Authors' contributions

Both authors conceived the convenience kinetics and carried out calculations. W.L. wrote the manuscript, E. K. identified the underlying molecular mechanism and revised the manuscript. Both authors read and approved the final manuscript.

## Supplementary Material

Additional file 1 The supplement file contains a list of the mathematical symbols used, the derivation of equation (22), and a detailed explanation of the sensitivity matrix Rθx
 MathType@MTEF@5@5@+=feaafiart1ev1aaatCvAUfKttLearuWrP9MDH5MBPbIqV92AaeXatLxBI9gBaebbnrfifHhDYfgasaacH8akY=wiFfYdH8Gipec8Eeeu0xXdbba9frFj0=OqFfea0dXdd9vqai=hGuQ8kuc9pgc9s8qqaq=dirpe0xb9q8qiLsFr0=vr0=vr0dc8meaabaqaciaacaGaaeqabaqabeGadaaakeaacqWGsbGudaqhaaWcbaacciGae8hUdehabaGaemiEaGhaaaaa@313C@. Click here for file
