# Supplementary material for: Bringing metabolic networks to life: convenience rate law and thermodynamic constraints
Source: Theor Biol Med Model. 2006 Dec 15;3:41. doi: 10.1186/1742-4682-3-41 (PMC1781438; doi:10.1186/1742-4682-3-41)
Supplement: Additional file 1 — The supplement file contains a list of the mathematical symbols used, the derivation of equation (22), and a detailed explanation of the sensitivity matrix Rθx MathType@MTEF@5@5@+=feaafiart1ev1aaatCvAUfKttLearuWrP9MDH5MBPbIqV92AaeXatLxBI9gBaebbnrfifHhDYfgasaacH8akY=wiFfYdH8Gipec8Eeeu0xXdbba9frFj0=OqFfea0dXdd9vqai=hGuQ8kuc9pgc9s8qqaq=dirpe0xb9q8qiLsFr0=vr0=vr0dc8meaabaqaciaacaGaaeqabaqabeGadaaakeaacqWGsbGudaqhaaWcbaacciGae8hUdehabaGaemiEaGhaaaaa@313C@. [file 1742-4682-3-41-S1.pdf]

Bringing metabolic networks to life: convenience rate law and thermodynamic constraints Supplementary material

Wolfram Liebermeister[1*], Edda Klipp[1]

[1]Computational Systems Biology, Max Planck Institute for Molecular Genetics,
Ihnestraße 63-73, 14195 Berlin, Germany

Email: Wolfram Liebermeister - lieberme@molgen.mpg.de; Edda Klipp - klipp@molgen.mpg.de;

*Corresponding author

List of mathematical symbols

Symbol	Meaning
$N = (n_{il})$	stoichiometric matrix
$W = (w_{li})$	regulation matrix
$\mathbf{c} = (c_i)$	vector of metabolite concentrations
$\mathbf{v} = (v_l)$	vector of reaction velocities
$\mathbf{k}$	vector of model parameters
$G_i^{(0)}$	Gibbs free energy of formation of metabolite i
k_i^{G}	energy constant of metabolite i
k_l^{V}	velocity constant of reaction l
k_{li}^{M}	Michaelis-Menten constant of reaction l and metabolite i
k_{li}^{A}	activation constant of reaction l and metabolite i
k_{li}^{I}	inhibition constant of reaction l and metabolite i
$\mathbf{k}^{\mathrm{eq}} = (k_l^{\mathrm{eq}})$	vector of equilibrium constants
$\mathbf{k}^{\mathrm{ind}}$	vector of independent equilibrium constants
$R_{\mathrm{ind}}^{\mathrm{eq}}$	matrix to compute $\mathbf{k}^{\mathrm{eq}}$ from $\mathbf{k}^{\mathrm{ind}}$
E_l	total enzyme concentration of reaction l
$k_{\pm l}^{\mathrm{cat}}$	maximal turnover rates (forward and backward) of reaction l
$v_{\pm l}^{\mathrm{max}}$	maximal velocities (forward and backward) of reaction l
θ	vector of system parameters (logarithmic)
$\mathbf{x}^*$	vector of kinetic data (logarithmic)
R_θ^x	matrix to predict $\mathbf{x}^*$ from θ

Derivation of equation (22)

From definition (20), we obtain

$$\ln k^{\mathrm{cat}}_{-l} \quad = \quad 2 \ln k^{\mathrm{V}}_{l} - \ln k^{\mathrm{cat}}_{+l}. \tag{1}$$

By inserting this into (17), equating to eqn. (18), and using definition (19), we obtain

$$-\sum_i n_{il} \;\ln k^{\mathrm{G}}_{i} = 2 \ln k^{\mathrm{cat}}_{+l} - 2 \ln k^{\mathrm{V}}_{l} + \sum_i n_{il} \ln k^{\mathrm{M}}_{li}, \tag{2}$$

which can be solved for

$$\ln k^{\mathrm{cat}}_{+l} = \ln k^{\mathrm{V}}_{l} - \frac{1}{2} \sum_i n_{il} \;(\ln k^{\mathrm{M}}_{li} + \ln k^{\mathrm{G}}_{i}). \tag{3}$$

Employing again the above eqn. (1), we obtain

$$\ln k^{\mathrm{cat}}_{-l} = \ln k^{\mathrm{V}}_{l} + \frac{1}{2} \sum_i n_{il} \;(\ln k^{\mathrm{M}}_{li} + \ln k^{\mathrm{G}}_{i}). \tag{4}$$

Taking the exponential of (3) and (4) leads directly to equation (22).

Computing the matrix R^x_θ: an example

The sensitivity matrix R^x_θ relates the measured kinetic parameters $\mathbf{x}^*$ to the independent system parameters θ. Both θ and $\mathbf{x}^*$ contain logarithmic values. We demonstrate the construction of R^x_θ with the following example network:

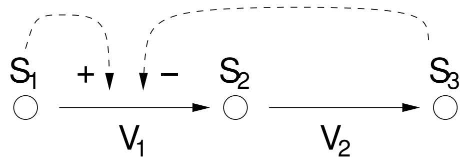

S_1 is a fixed metabolite and activates reaction V_1, while S_3 inhibits reaction V_1 (dashed arrows). The stoichiometric matrix and the regulation matrix read

$$N \quad = \quad \begin{pmatrix} -1 & 0 \\ 1 & -1 \\ 0 & 1 \end{pmatrix}, \quad W = \begin{pmatrix} 1 & 0 & -1 \\ 0 & 0 & 0 \end{pmatrix}. \tag{5}$$

We include the enzyme concentrations E_l into the model parameters and assume that all system parameters as well as equilibrium constants k^{eq}_l, turnover rates $k^{\mathrm{cat}}_{\pm l}$, and maximal velocities $v^{\mathrm{max}}_{\pm}$ can be measured. The matrix R^x_θ reads (dots represent zero elements):

	k_1^{G}	k_2^{G}	k_3^{G}	k_1^{V}	k_2^{V}	k_{11}^{M}	k_{12}^{M}	k_{22}^{M}	k_{23}^{M}	k_{11}^{A}	k_{13}^{I}	E_1	E_2
k_1^{G}	1	·	·	·	·	·	·	·	·	·	·	·	·
k_2^{G}	·	1	·	·	·	·	·	·	·	·	·	·	·
k_3^{G}	·	·	1	·	·	·	·	·	·	·	·	·	·
k_1^{V}	·	·	·	1	·	·	·	·	·	·	·	·	·
k_2^{V}	·	·	·	·	1	·	·	·	·	·	·	·	·
k_{11}^{M}	·	·	·	·	·	1	·	·	·	·	·	·	·
k_{12}^{M}	·	·	·	·	·	·	1	·	·	·	·	·	·
k_{22}^{M}	·	·	·	·	·	·	·	1	·	·	·	·	·
k_{23}^{M}	·	·	·	·	·	·	·	·	1	·	·	·	·
k_{11}^{A}	·	·	·	·	·	·	·	·	·	1	·	·	·
k_{13}^{I}	·	·	·	·	·	·	·	·	·	·	1	·	·
E_1	·	·	·	·	·	·	·	·	·	·	·	1	·
E_2	·	·	·	·	·	·	·	·	·	·	·	·	1
k_1^{eq}	1	-1	·	·	·	·	·	·	·	·	·	·	·
k_2^{eq}	·	1	-1	·	·	·	·	·	·	·	·	·	·
k_{+1}^{cat}	$-\frac{1}{2}$	$\frac{1}{2}$	·	1	·	$\frac{1}{2}$	$-\frac{1}{2}$	·	·	·	·	·	·
k_{+2}^{cat}	·	$-\frac{1}{2}$	$\frac{1}{2}$	·	1	·	·	$\frac{1}{2}$	$-\frac{1}{2}$	·	·	·	·
k_{-1}^{cat}	$\frac{1}{2}$	$-\frac{1}{2}$	·	1	·	$-\frac{1}{2}$	$\frac{1}{2}$	·	·	·	·	·	·
k_{-2}^{cat}	·	$\frac{1}{2}$	$-\frac{1}{2}$	·	1	·	·	$-\frac{1}{2}$	$\frac{1}{2}$	·	·		
$v_{+1}^{\max}$	$-\frac{1}{2}$	$\frac{1}{2}$	·	1	·	$\frac{1}{2}$	$-\frac{1}{2}$	·	·	·	·	1	·
$v_{+2}^{\max}$	·	$-\frac{1}{2}$	$\frac{1}{2}$	·	1	·	·	$\frac{1}{2}$	$-\frac{1}{2}$	·	·	·	1
$v_{-1}^{\max}$	$\frac{1}{2}$	$-\frac{1}{2}$	·	1	·	$-\frac{1}{2}$	$\frac{1}{2}$	·	·	·	·	1	·
$v_{-2}^{\max}$	·	$\frac{1}{2}$	$-\frac{1}{2}$	·	1	·	·	$-\frac{1}{2}$	$\frac{1}{2}$	·	·	·	1

Its upper part corresponds to direct measurements of the system parameters; it is just an identity matrix. The lower part shows how the equilibrium constants, turnover rates, and maximal velocities depend on the system parameters, based on $\ln \mathbf{k}^{\mathrm{eq}} = -N^{\mathrm{T}} \mathbf{k}^{\mathrm{G}}$ (compare eqs. (18) and (22)). The sensitivity matrix R_θ^x can be written in block matrix form

	k^{G}	k^{V}	k^{M}	k^{A}	k^{I}	E
k^{G}	$\mathbf{I}$	·	·	·	·	·
k^{V}	·	$\mathbf{I}$	·	·	·	·
k^{M}	·	·	$\mathbf{I}$	·	·	·
k^{A}	·	·	·	$\mathbf{I}$	·	·
k^{I}	·	·	·	·	$\mathbf{I}$	·
E	·	·	·	·	·	$\mathbf{I}$
k^{eq}	$-N^{\mathrm{T}}$	·	·	·	·	·
k_{+}^{cat}	$\frac{1}{2}N^{\mathrm{T}}$	$\mathbf{I}$	$-\frac{1}{2}Z$	·	·	·
k_{-}^{cat}	$-\frac{1}{2}N^{\mathrm{T}}$	$\mathbf{I}$	$\frac{1}{2}Z$	·	·	·
$v_{+}^{\max}$	$\frac{1}{2}N^{\mathrm{T}}$	$\mathbf{I}$	$-\frac{1}{2}Z$	·	·	$\mathbf{I}$
$v_{-}^{\max}$	$-\frac{1}{2}N^{\mathrm{T}}$	$\mathbf{I}$	$\frac{1}{2}Z$	·	·	$\mathbf{I}$

where each column of the matrix Z

$$\begin{array}{c} \\ v_1 \\ v_2 \end{array} \begin{array}{c} \begin{array}{cccc} k_{11}^{\mathrm{M}} & k_{12}^{\mathrm{M}} & k_{22}^{\mathrm{M}} & k_{23}^{\mathrm{M}} \end{array} \\ \left(\begin{array}{cccc} -1 & 1 & \cdot & \cdot \\ \cdot & \cdot & -1 & 1 \end{array} \right) \end{array}$$

corresponds to one of the k_{li}^{M} values; it contains the stoichiometric coefficient for the corresponding l^{th} reaction and zeroes for all other reactions. For parameter estimation, we only consider the kinetic parameters that have been measured and collected in the vector $\mathbf{x}^*$. Hence, we use an incomplete sensitivity matrix, built only from those rows of R_θ^x that correspond to the measured parameters.
